# Flexible Edible Materials from Extrusion-Plasticized Modified Cassava Starches with Maltol and Ethyl Maltol: Molecular Interactions, Mechanical Properties, and Antioxidant and Antimicrobial Activities

**DOI:** 10.3390/foods15193393

**Published:** 2026-09-23

**Authors:** Khwanchat Promhuad, Phatthranit Klinmalai, Shyam S. Sablani, Jong-Whan Rhim, Nathdanai Harnkarnsujarit

**Affiliations:** 1 Department of Packaging and Materials Technology, Faculty of Agro-Industry, Kasetsart University, 50 Ngam Wong Wan Rd., Latyao, Chatuchak, Bangkok 10900, Thailand; khwanchatpromhuad@gmail.com; 2 Faculty of Agro-Industry, Chiang Mai University, Samut Sakhon 74000, Thailand; phatthranit.k@cmu.ac.th; 3 Department of Biological System Engineering, Washington State University, Pullman, WA 99164, USA; ssablani@wsu.edu; 4 BioNanocomposite Research Center, Department of Food and Nutrition, Kyung Hee University, Seoul 02447, Republic of Korea; 5 Center for Advanced Studies for Agriculture and Food (CASAF), Kasetsart University Institute for Advanced Studies (KUIAS), Kasetsart University, 50 Ngam Wong Wan Rd., Latyao, Chatuchak, Bangkok 10900, Thailand

**Keywords:** extrusion, maltol, starch–flavor interactions, antioxidant, antimicrobial, packaging

## Abstract

This study systematically investigated how cassava starch modification chemistry and hydroxypropyl substitution level influence the structure and functional performance of extrusion-plasticized edible materials containing maltol or ethyl maltol. Native starch, acetylated starch, octenyl succinate-modified starch, and hydroxypropylated starches with low, medium, and high substitution levels were incorporated with 3% maltol or ethyl maltol and processed by twin-screw extrusion. The resulting materials were characterized for microstructure, molecular environment, crystalline organization, thermal behavior, mechanical properties, antioxidant activity, and antimicrobial performance. FTIR and NMR analyses showed changes in the molecular environment after incorporation of the maltol derivatives, while XRD revealed formulation-dependent differences in structural ordering. Mechanical responses were strongly matrix-dependent, with tensile strength, elongation at break, and Young’s modulus reaching up to 3.56 MPa, 552%, and 108 MPa, respectively. DPPH radical-scavenging activity increased with extraction time and reached approximately 80–82% after 5 h in the most active formulations. Antibacterial activity was selective, with the largest inhibition zone against *Bacillus subtilis* reaching 3.53 mm, whereas no inhibition zones were detected against *Staphylococcus aureus* or *Escherichia coli*. Antifungal activity was more pronounced, with maximum inhibition zones of 7.39 mm against *Aspergillus niger* and 0.55 mm against *Penicillium* sp.; no distinct inhibition zone was observed against *Rhizopus* sp., although delayed sporulation was observed in some maltol-containing samples. These findings demonstrate that starch modification chemistry and substitution level govern the balance among structural organization, mechanical behavior, antioxidant activity, and selective antimicrobial performance of extrusion-plasticized cassava starch matrices containing maltol derivatives.

## 1. Introduction

Starch is a renewable, biodegradable, and food-compatible biopolymer with considerable potential for the development of edible materials and structured food matrices. Cassava starch is particularly attractive because of its wide availability, relatively high purity, neutral taste, and ability to form continuous polymeric matrices. Nevertheless, native starch generally exhibits limited processability, brittleness at low moisture contents, and marked sensitivity to environmental humidity. These limitations restrict its use in flexible edible materials and necessitate structural modification and plasticization to improve molecular mobility and mechanical performance [1,2,3,4,5]. Starch consists primarily of amylose and amylopectin. Cassava starch is generally rich in amylopectin, which accounts for approximately 80% of the starch. Amylose consists predominantly of linear α-(1→4)-linked glucan chains, whereas amylopectin has a highly branched structure containing α-(1→4)-linked chains connected through α-(1→6) branch points. Amylopectin is the principal contributor to the semicrystalline lamellar organization of starch granules, while amylose is distributed mainly within less ordered regions. Consequently, changes in amylose–amylopectin organization, hydrogen bonding, and chain packing can strongly influence gelatinization, plasticization, crystallinity, and the response of starch to thermomechanical processing.

Extrusion provides a continuous and scalable means of converting granular starch into a processable polymeric matrix through the combined effects of heat, shear, pressure, and moisture. In conventional food extrusion, starch gelatinization and rapid water evaporation at the die commonly produce expanded, porous products that become rigid and glassy after cooling. In contrast, extrusion in the presence of an appropriate plasticizer can disrupt inter- and intramolecular hydrogen bonding, increase starch-chain mobility, and promote the formation of a dense, cohesive material with greater flexibility [2,5]. The properties of such extrusion-plasticized starch matrices depend not only on processing conditions and plasticizer content but also on the molecular structure of the starch. Differences in chain organization, substituent chemistry, and degree of modification may therefore govern the ability of starch to interact with incorporated low-molecular-weight compounds.

Chemical modification provides a means of tailoring starch polarity, chain association, water affinity, and compatibility with other ingredients. Acetylation and hydroxypropylation introduce substituent groups that reduce interactions among starch hydroxyl groups and may increase chain mobility, whereas octenyl succinylation introduces both hydrophilic and hydrophobic moieties, imparting amphiphilic characteristics to the starch [1,2,3]. The extent of substitution can further influence molecular packing, plasticizer compatibility, mechanical behavior, and the retention or release of active compounds. Although these modified starches are widely used individually as food ingredients, their comparative behavior during extrusion plasticization has received limited attention. In particular, it remains unclear how the type and level of starch modification affect interactions with flavor-active compounds and how these interactions are translated into the microstructure, mechanical properties, and functional activities of the resulting edible materials.

Maltol (3-hydroxy-2-methyl-4H-pyran-4-one) and ethyl maltol (2-ethyl-3-hydroxy-4H-pyran-4-one) are structurally related flavor enhancers characterized by sweet, caramel-like aromas. Ethyl maltol is an ethyl-substituted analog of maltol and generally exhibits greater flavor-enhancing potency. In addition to their established flavor functions, the hydroxypyrone structures of these compounds enable metal-ion chelation and free radical scavenging, providing potential antioxidant activity. Antimicrobial effects against selected bacteria and fungi have also been reported, although maltol and ethyl maltol are used primarily as flavoring substances rather than as conventional preservatives [3,6,7,8,9]. Their incorporation into an edible starch matrix may therefore provide multiple functions, including flavor enhancement, antioxidant activity, and microbial growth inhibition. However, these functions may depend strongly on the molecular environment within the matrix, as starch–compound interactions can affect the distribution, accessibility, retention, and release of the incorporated compounds.

The thermomechanical conditions encountered during extrusion may further influence these interactions. Heat and shear disrupt the native granular and semicrystalline organization of starch, expose hydroxyl groups, and promote contact among starch chains, the plasticizer, and incorporated compounds. Maltol and ethyl maltol contain hydroxyl and carbonyl functionalities capable of participating in non-covalent interactions with starch, particularly through hydrogen bonding [3,9]. Such interactions may affect molecular packing, matrix continuity, chain mobility, and stress transfer within the plasticized material. At the same time, immobilization or association of maltol derivatives within the starch matrix may alter their accessibility and, consequently, their measured antioxidant and antimicrobial activities. Understanding these molecular relationships is therefore essential for controlling both the structural and functional performance of the resulting materials.

Previous studies have examined interactions between ethyl maltol and starch under heat–moisture treatment and have demonstrated that starch–ethyl maltol association can alter starch structure and functionality [3,9]. The effects of ethyl maltol on selected starch-based active materials have also begun to receive attention. Nevertheless, a systematic comparison of maltol and ethyl maltol in extrusion-plasticized cassava starches possessing different substituent chemistries and modification levels remains lacking. More specifically, limited information is available on how acetyl, octenyl succinate, and hydroxypropyl groups influence the molecular association of maltol derivatives with starch under thermomechanical processing, or how these associations determine the microstructure, tensile behavior, and antioxidant and antimicrobial activities of dense, flexible extrudates. Establishing these relationships is important for the rational selection of modified starch matrices for multifunctional edible materials.

Accordingly, this study investigated acetylated and octenyl succinate-modified cassava starches, together with hydroxypropylated cassava starches representing low, medium, and high substitution levels. Each starch was plasticized by twin-screw extrusion and incorporated with either maltol or ethyl maltol. Molecular and microstructural characteristics were examined using Fourier-transform infrared spectroscopy, nuclear magnetic resonance spectroscopy, and scanning electron microscopy, and their relationships with the mechanical, antioxidant, and antimicrobial properties of the extrudates were evaluated. It was hypothesized that differences in substituent chemistry among the modified starch matrices would influence their non-covalent interactions with the plasticizer and maltol derivatives. Within the hydroxypropylated starch series, the level of substitution was further hypothesized to regulate starch-chain association, matrix organization, and the resulting mechanical and bioactive properties. This comparative approach provides insight into the effects of both starch modification chemistry and hydroxypropyl substitution level on the structure–interaction–property relationships of flexible, extrusion-plasticized edible materials.

## 2. Materials and Methods

### 2.1. Materials and Processing

#### 2.1.1. Materials

Food-grade ethyl maltol (2-Ethyl-3-hydroxy-4H-pyran-4-one) and food-grade maltol (3-Hydroxy-2-methyl-4H-pyran-4-one) were used as flavor enhancer powders (Sigma-Aldrich, Buchs, Switzerland). Native starch (NT), acetylated starch (AC; supplier-specified DS range: 0.01–0.03), octenyl succinate-modified starch (OS; supplier-specified DS range: 0.01–0.03), and three commercial hydroxypropylated starch grades designated by the supplier as low (HL; <0.07), medium (HM; 0.07–0.09), and high (HH; >0.09) substitution were obtained from Siam Modified Starch Co., Ltd., Pathum Thani, Thailand. These values represent supplier specification ranges rather than batch-specific experimentally determined substitution values.

#### 2.1.2. Starch Extrusion Processing

Ethyl maltol (E) or maltol (M) was mixed with starch at 3% *w*/*w* of total solids (Table 1). The powder mixtures were supplemented with glycerol at a weight ratio of 100:35 solids and mixed in the mixing bowl of a dough mixer (SC-236A, Stelang, Foshan, China) for at least 10 min. The mixtures were extruded using a twin-screw extruder (LVF-2202–130, L/D ratio = 48, screw diameter = 20 mm, Labtech Engineering Co. Ltd., Samut Prakan, Thailand). The temperature profile from the feed zone to the die zone was set to 80, 85, 115, 125, 130, 135, 140, 140, 145, 150, 150, and 155 °C, respectively, at a screw speed of 200 RPM. The starch extrudates were kept in heat-sealed aluminum bags in a humidity-controlled environment at 25 °C and 50% relative humidity for a minimum of 48 h until further experiments.

### 2.2. Characterization of the Starch Extrusion

#### 2.2.1. Scanning Electron Microscopy (SEM)

The microstructure of the extruded starch was examined using a scanning electron microscope (FEI Quanta 450, Thermo Fisher Scientific, Waltham, MA, USA) at 10 kV. The starch extrudates were fractured in liquid nitrogen and then coated with gold using a sputter coater (SPI-Module, Structure Probe, Inc., West Chester, PA, USA).

#### 2.2.2. Optical Properties

The amount of light transmission was measured using an Evolution 300 UV-Vis Spectrophotometer (Thermo Fisher Scientific, Waltham, MA, USA) UV-visible spectrometer. Starch extrudates were divided into 4 × 4 rectangles and scanned at 600 nm/min across wavelengths from 200 to 800 nm. Three independent specimens were analyzed for each formulation (n = 3). Each specimen was measured twice as a technical replicate, and the duplicate readings were averaged to obtain one value per specimen. The reported results therefore represent the mean ± standard deviation of three independent specimens.

#### 2.2.3. Fourier-Transform Infrared Spectroscopy (FTIR)

Attenuated total reflectance Fourier-transform infrared spectroscopy (ATR-FTIR, Tensor 27 FTIR, Bruker Optics GmbH, Ettlingen, Germany) was used to measure the infrared absorption spectra of the starch extrudates. Spectra were collected in three replicates at a resolution of 4 cm^−1^ and averaged over 64 scanning cycles. The extruded starch was scanned over wavenumbers ranging from 500 to 4000 cm^−1^.

#### 2.2.4. Nuclear Magnetic Resonance (NMR) Spectroscopy

An Ascend TM 600/Advance III HD (Bruker, Fällanden, Switzerland) was used to record the ^1^H NMR spectra. The extruded starch (25 mg) was dissolved in DMSO and run at 600 MHz. Tetramethylsilane was used as the reference, and chemical shifts were reported in parts per million (ppm).

#### 2.2.5. Matrix-Assisted Laser Desorption/Ionization Time-of-Flight Mass Spectrometer (MALDI-TOF MS)

All samples were analyzed using a matrix-assisted laser desorption ionization-time-of-flight mass spectrometry (MALDI-TOF MS) ID-Plus (Shimadzu, Kyoto, Japan) in reflectron mode (*m*/*z* = 0–6000). Samples (0.5 μL) were overlaid with 0.5 µL of a 2,5-dihydroxybenzoic acid matrix solution (DHB; 10 mg/mL in acetonitrile/0.1% trifluoroacetic acid 1:1) and air-dried at 24–27 °C.

#### 2.2.6. X-Ray Diffraction (XRD)

The crystallinity of the extruded starch was assessed using an X-ray diffractometer (Diffractometer D8, Bruker AXS, Karlsruhe, Germany). The starch extrudates were scanned over a 2° range from 4° to 40° at 40 kV and 40 mA. The step size was 0.02°, and the scanning speed was 0.8°/s.

#### 2.2.7. Thermal Degradation Behavior of the Starch Extrudates

The thermal stability of the extruded starch was evaluated using simultaneous thermogravimetric analysis (TGA) and differential scanning calorimetry (DSC) (TGA/DSC3+, Mettler Toledo, Greifensee, Switzerland). Samples (5–10 mg) were weighed into alumina crucibles and heated at 10 °C/min from 30 to 700 °C in a nitrogen atmosphere. Derivative thermogravimetry (DTG) curves were generated using Mettler STARe version 10.00 software.

#### 2.2.8. Mechanical Properties

Tensile strength (TS), elongation at break (EB), and Young’s modulus (YM) were measured using an Instron Universal Testing Machine (Model 5965, Instron, Norwood, MA, USA) in accordance with ASTM D882-10 [10]. The 150 × 25 mm samples were conditioned at 50% relative humidity for 48 h prior to measurement. Data from ten replicate samples were then averaged.

#### 2.2.9. Antioxidant Activities of the Starch Extrudates

The diphenyl picryl hydrazine (DPPH) radical is a commonly used assay for assessing the antioxidant activity of substances or potential hydrogen donors by scavenging free radicals. A UV-Vis spectrophotometer (Spectronic Genesys 10 UV, Thermo Fisher Scientific, USA) was used to measure the scavenging activity of 2,2-diphenyl-1-picrylhydrazyl (DPPH) radicals using extruded starch. Ten milligrams of DPPH were dissolved in one thousand milliliters of ethanol to create a DPPH solution (10 ppm DPPH). Next, 10 mL of the prepared DPPH solution was added to vials containing 50 mg of each sample. The solution was left to sit at room temperature in the dark for 0, 1, 2, 3, 4, and 5 h. A UV-Vis spectrophotometer was then used to measure the extracted solution’s absorbance at 517 nm. Spectrophotometric measurements were performed using pure ethanol (99.99% purity) as the blank solution. Three samples of each extruded starch mix were examined. The following Equation (1) was used to determine each active edible extruded starch’s antioxidant activity.

(1) $$DPPH\;scavenging\;activity\left(\%\right)=\frac{A_{DPPH}-A_{sample}}{A_{DPPH}}\times 100$$

where *A_DPPH_* and *A_sample_* are the absorbance values of the *DPPH* solution and the sample extract at 517 nm after storage [11].

### 2.3. Antimicrobial Activities of the Starch Extrudates

#### 2.3.1. Antifungal Activities of the Starch Extrudates

The concentration of fungi (*Aspergillus niger*, *Penicillium* sp., and *Rhizopus* sp.) was adjusted to 10^6^ spores/mL using a Neubauer hemocytometer (Marienfeld, Karlsruhe, Germany). One milliliter of the fungal suspension was spread onto the surface of potato dextrose agar (PDA) using a triangle spreader. Both sides of the extruded starch samples were disinfected by placing them under UV lamps in a laminar flow cabinet (Lab Micro Co., Ltd., Bangkok, Thailand) for 7 min. A sterilized starch extrudate disk was then placed on the surface of the PDA media containing fungi. Petri plates were sealed with Parafilm and incubated at 25 °C for 5 days. The control group consisted of PDA media plates without extruded starch samples.

#### 2.3.2. Antibacterial Activities of the Starch Extrudates

The initial inoculum levels of *B. subtilis*, *S. aureus*, and *E. coli* in peptone at 37 °C were 8.09, 6.99, and 8.69 log CFU/g, respectively. These strains were chosen to represent a range of foodborne pathogens and spoilage organisms. *S. aureus* and *B. subtilis* are common contaminants in ready-to-eat foods and bakery products, while *E. coli* serves as an indicator of hygiene and safety across a broad spectrum of food categories. The bacteria were used to prepare the inoculum by incubating in nutrient broth at 37 °C for 24 h. The suspension (1 mL) was dispensed onto the surface of nutrient agar (NA) using a triangular spreader. The Petri dishes were covered with Parafilm and incubated at 37 °C for 24 h [12].

### 2.4. Statistical Analysis

Analysis of variance (ANOVA) at a 95% confidence level was used to determine significant differences in starch extrudate attributes using the statistics program SPSS 27 (SPSS Inc., Chicago, IL, USA), while Duncan’s multiple range test was used to detect major differences between mixes.

## 3. Results

### 3.1. Physical Properties

#### 3.1.1. Microstructures of the Starch Extrudates

The surface and cross-sectional microstructures of the starch extrudates containing maltol or ethyl maltol are shown in Figure 1A. Among the hydroxypropylated starch samples, the extrudates with a high substitution level and without added maltol or ethyl maltol exhibited smoother surfaces than those prepared from hydroxypropylated starch with a low substitution level. This difference was likely associated with the increased number of hydroxypropyl groups, which reduced intermolecular associations among the starch chains and increased chain mobility and free volume. Consequently, the higher substitution level may have enhanced plasticization and promoted the formation of a more continuous matrix during extrusion. The addition of maltol or ethyl maltol further increased surface smoothness and reduced surface roughness. These compounds may have facilitated starch plasticization and granule disruption during extrusion, thereby promoting interactions between the starch matrix and the incorporated maltol derivatives [3].

The SEM micrographs of the starch extrudates without added maltol or ethyl maltol showed small particles dispersed throughout the cross-sectional matrices. These particles were tentatively attributed to residual starch granules or incompletely disrupted granular structures and showed a tendency to form aggregates or clusters [13]. Although maltol and ethyl maltol generally improved the smoothness of the external surfaces, irregular structures remained visible in the cross-sections of some extrudates containing 3% maltol or ethyl maltol. These internal heterogeneities may represent residual granular structures, aggregated domains, or crystalline regions within the extruded matrices. However, their crystalline nature cannot be confirmed from SEM observations alone. Promhuad et al. (2022) [3] similarly reported heterogeneous structures in cassava starch films containing 1–5% maltol, as observed by SEM.

#### 3.1.2. Light Transmissions

The starch extrudates without added maltol or ethyl maltol shown in Figure S1A (Supplementary Materials) were colorless, whereas those containing 3% maltol or ethyl maltol developed an orange tint. The color development was more pronounced in the ethyl maltol-containing samples than in those containing maltol. The relatively uniform coloration was consistent with the distribution of the maltol derivatives throughout the starch matrices, although color observations alone cannot confirm molecular miscibility.

The light transmittance of the extrudates containing maltol or ethyl maltol decreased over the wavelength range of 300–500 nm (Figure S1B, Supplementary Materials). This decrease indicates enhanced attenuation of near-ultraviolet and short-wavelength visible light rather than selective blocking of ultraviolet A radiation alone. The lower transmittance may be attributed to light absorption by colored compounds formed or retained during extrusion, together with light scattering caused by crystalline regions, residual starch granules, or other structural heterogeneities within the matrices. Accordingly, the maltol- and ethyl maltol-containing extrudates appeared more turbid and showed lower optical clarity than the corresponding control samples [14]. The lower transmittance in the UV and short-wavelength visible regions is also relevant to the potential food-protective function of these materials. UV radiation contains relatively high-energy photons capable of initiating or accelerating photochemical reactions in light-sensitive food constituents. Exposure to UV and visible light can promote oxidative deterioration of lipids and degradation of vitamins, pigments, flavors, and other nutrients, thereby reducing food quality and shelf life.

### 3.2. Crystalline Morphology

The X-ray diffraction patterns of the starch extrudates are shown in Figure 1B. The patterns contained both broad and distinct diffraction features, indicating that the extrudates retained different degrees of ordered or semicrystalline structure after extrusion. A distinct peak at approximately 2θ = 24° was observed in all samples. Within the hydroxypropylated starch series, the extrudates prepared from starch with a low substitution level exhibited relatively sharp peaks at approximately 2θ = 13.0°, 18.2°, and 19.7°. The intensity of these peaks decreased as the hydroxypropyl substitution level increased. The introduction of a greater number of bulky hydroxypropyl groups may have disrupted starch-chain packing and inhibited the formation of ordered crystalline regions. At the same time, water absorption and plasticization increased molecular mobility during extrusion. Although enhanced mobility can facilitate chain rearrangement during cooling or subsequent conditioning, extensive substitution may restrict the regular chain alignment required for crystallization.

The crystalline diffraction peaks associated with maltol were more pronounced than those observed in the ethyl maltol-containing extrudates. Starch extrudates containing maltol showed a sharp diffraction peak at approximately 2θ = 14.7°, suggesting the presence of residual or recrystallized maltol domains within the starch matrix. In contrast, no distinct diffraction peak attributable to crystalline ethyl maltol was detected in the ethyl maltol-containing extrudates. This absence may indicate that ethyl maltol was present in a less ordered or more molecularly dispersed form, or that its crystalline fraction was below the detection limit of the XRD measurement. Steric effects associated with the ethyl substituent may hinder efficient molecular packing and crystal nucleation [15]. In addition, stronger associations between ethyl maltol and the starch matrix may restrict molecular aggregation and recrystallization. However, the absence of a diffraction peak alone does not distinguish conclusively between molecular dispersion, amorphization, and the formation of very small or low abundance crystalline domains.

### 3.3. Interaction Between Starch and Ethyl Maltol or Maltol

#### 3.3.1. Fourier-Transform Infrared (FTIR) Spectroscopy

The FTIR spectra of the extruded starch matrices were dominated by absorption bands originating from the polysaccharide backbone, glycerol, and the incorporated maltol derivatives. Starch consists mainly of amylose and amylopectin, with cassava starch typically containing approximately 80% amylopectin. The highly branched amylopectin structure contributes substantially to the semicrystalline organization of starch and contains both α-(1→4) and α-(1→6) glycosidic linkages. Therefore, changes in the FTIR spectrum after extrusion and chemical modification can reflect alterations in hydrogen-bonding environments and short-range organization of the polysaccharide chains rather than changes associated exclusively with maltol or ethyl maltol.

The FTIR spectra of the starch extrudates, particularly in the region of 1150–1750 cm^−1^, are shown in Figure 2A. The spectra of the extrudates containing maltol or ethyl maltol showed absorption bands associated with the starch matrix and the incorporated maltol derivatives. Bands in the region of 1300–1500 cm^−1^ were associated mainly with C–H bending vibrations, whereas the band at approximately 1562 cm^−1^ was attributed to vibrations of the conjugated pyrone ring rather than to benzene-ring vibrations [16]. Maltol and ethyl maltol showed characteristic absorption bands at approximately 1201 and 1257 cm^−1^, associated with C–O stretching, and at approximately 1562, 1620, and 1654–1657 cm^−1^, associated with vibrations of the conjugated ring and carbonyl groups.

The intensities of these bands generally increased in the extrudates containing maltol, and more distinct peaks were observed in this region. The absorption bands at approximately 1620 and 1654–1657 cm^−1^ were attributed primarily to C=O stretching vibrations. Maltol may occur in different crystalline arrangements, which can produce differences in the molecular environment and vibrational energy of its carbonyl group. The presence of more than one carbonyl-related band may therefore be associated with different molecular environments or crystalline forms of maltol. Nevertheless, confirmation of specific maltol polymorphs would require supporting crystallographic or thermal evidence.

The bands at approximately 1201 and 1257 cm^−1^ were associated with C–O vibrations of maltol and ethyl maltol [9]. Absorption in this region may also overlap with C–O–C and C–O stretching vibrations of the glycosidic structure of starch [4]. Changes in band position, shape, and relative intensity after incorporation of maltol or ethyl maltol indicated an altered local molecular environment within the extruded starch matrices. However, because several bands in this region overlap with C–O and C–O–C vibrations of the starch backbone, these spectral changes cannot be assigned uniquely to a specific starch–maltol interaction. They may reflect differences in molecular packing, hydrogen-bonding environment, and/or overlap between starch and maltol-derivative vibrations. Therefore, FTIR was interpreted qualitatively and was not used to confirm a specific hydrogen-bonding mechanism.

#### 3.3.2. Nuclear Magnetic Resonance (NMR) Spectroscopy

^1^H-NMR spectra of the starch extrudates are shown in Figure 2B. The spectra of all starch samples contained signals associated with the anhydroglucose units of starch, together with signals from the solvent and residual water. The signal at δ 2.50 ppm was attributed to residual dimethyl sulfoxide (DMSO-d_6_), while overlapping starch-ring proton signals were observed mainly at δ 3.0–4.0 ppm. The anomeric H1 signal and hydroxyl proton signals appeared primarily within the region of δ 4.3–5.6 ppm. The exact positions of the hydroxyl proton signals may vary depending on the starch structure, water content, concentration, and hydrogen-bonding environment.

The hydroxypropylated starches showed a characteristic doublet at approximately δ 1.1–1.2 ppm, assigned to the methyl protons of the hydroxypropyl groups. The acetylated starch showed a signal at approximately δ 2.0–2.1 ppm, corresponding to the methyl protons of the acetyl groups [17]. The signal at approximately δ 0.8 ppm in the octenyl succinate-modified starch was assigned to the terminal methyl protons of the octenyl succinate substituent.

Following the incorporation of 3% maltol or ethyl maltol, additional characteristic signals were detected. Maltol showed a methyl proton signal at approximately δ 2.1 ppm, whereas ethyl maltol showed signals associated with its ethyl group, including the terminal methyl protons at approximately δ 1.1 ppm. Signals at approximately δ 6.3 and 8.0 ppm were attributed to the two nonequivalent protons of the conjugated pyrone ring [18]. These signals confirmed the presence of maltol or ethyl maltol in the extruded starch matrices. Differences in their chemical shifts, signal widths, or intensities relative to those of the individual compounds may indicate changes in the local molecular environment arising from non-covalent associations with starch. The signals should not be assigned to O-xylene because neither maltol nor ethyl maltol contains a xylene or benzene structure.

The principal starch signal at approximately δ 5.106 ppm in the extrudates without maltol or ethyl maltol was further examined, as shown in Figure S2 (Supplementary Materials). Based on the correlation spectrum, signals from adjacent protons in the starch structure were assigned to H1, H2, H3, H4, H5, and the two H6 protons at δ 5.11, 3.31, 3.66, 3.35, 3.59, and 3.66/3.56 ppm, respectively. Additional signals at approximately δ 5.51, 5.40, and 4.58 ppm were assigned to OH2, OH3, and OH6, respectively.

The relative intensity of the H1 signal in the starch extrudates is summarized in Figure S2 (Supplementary Materials). Changes in the profiles of the starch proton signals were observed following incorporation of maltol or ethyl maltol. Because the spectra were not acquired as quantitative NMR measurements, differences in absolute or relative peak intensity were not interpreted quantitatively. The NMR spectra were therefore used primarily for signal assignment and qualitative evaluation of changes in the local molecular environment. If the spectra were normalized using the same reference and processing conditions, this change may reflect alterations in the local chemical environment, molecular mobility, or signal overlap caused by the incorporation of the maltol derivatives. Changes in the positions and profiles of the starch proton signals further suggest modifications in electron shielding and hydrogen-bonding environments. Together with the FTIR results, these observations indicate changes in the local molecular environment following incorporation of maltol or ethyl maltol; however, the present spectroscopic data do not establish a specific intermolecular interaction mechanism.

#### 3.3.3. Matrix-Assisted Laser Desorption/Ionization Time-of-Flight Mass Spectrometer (MALDI-TOF MS)

Figure S3 (Supplementary Materials) shows the MALDI-TOF mass spectra of the starch samples. The spectra were characterized by a series of closely spaced signals within the detectable mass range, indicating the presence of starch-derived molecular species with different chain lengths, degrees of modification, or ion adducts. No distinct high molecular weight starch components were detected under the analytical conditions used. This observation should not be interpreted as the absence of high molecular weight starch chains because MALDI ionization and detection can be strongly influenced by molecular size, sample solubility, matrix selection, fragmentation, and ion suppression. The detected signals therefore represented primarily the fraction of the starch samples that could be ionized and detected under the selected conditions.

The modified starches exhibited detectable mass distributions broadly similar to that of native starch, although additional or shifted signals associated with their substituent groups may have been present. This similarity suggests that the detectable starch-derived fractions had comparable mass profiles; however, it does not demonstrate that the overall molecular-weight distributions of the native and modified starches were identical. Determination of the complete molecular weight distribution would require a complementary technique, such as size exclusion chromatography.

For the starch extrudates containing maltol or ethyl maltol, additional signals were observed at *m*/*z* values corresponding to the incorporated compounds or their ion adducts. These signals confirmed that maltol and ethyl maltol remained detectable after extrusion and were present in the corresponding starch materials. Nevertheless, MALDI-TOF MS alone cannot establish whether these compounds were molecularly dispersed, physically entrapped, or non-covalently associated with the starch matrix. The MALDI-TOF results therefore provided supporting evidence of compound retention after extrusion, while the nature of starch–compound interactions was interpreted in combination with the FTIR and NMR results.

### 3.4. Thermal Degradation Behavior of the Starch Extrudates

The thermogravimetric (TGA) and derivative thermogravimetric (DTG) curves used to evaluate the thermal degradation behavior of the starch extrudates are shown in Figure 3A and Figure 3B, respectively. An initial mass loss of approximately 0.5–1.8% occurred between 50 and 100 °C and was attributed mainly to the evaporation of free and weakly bound water from the starch matrices [5]. The relatively low mass loss in this region indicated that only limited residual moisture was present in the conditioned extrudates.

Following moisture loss, minor mass changes began at approximately 230 °C. These changes were associated with the initial thermal decomposition of low molecular weight components, including glycerol, maltol, and ethyl maltol, together with the onset of degradation of the starch matrix [9,19,20]. Maltol and ethyl maltol do not contain O-glycosidic bonds; therefore, their degradation should not be attributed to the cleavage of such bonds. Cleavage of glycosidic linkages is associated with thermal degradation of the starch chains.

The starch extrudates subsequently underwent substantial thermal degradation, resulting in an overall mass loss of approximately 90%. The DTG curves (Figure 3B) showed overlapping degradation events rather than completely independent decomposition stages. The lower-temperature region, beginning at approximately 230 °C, was associated primarily with the volatilization or decomposition of glycerol and the maltol derivatives, together with the initial degradation of starch. The principal degradation region, extending approximately from 230 to 400 °C, was attributed predominantly to the cleavage of glycosidic linkages, dehydration, depolymerization, and decomposition of the starch backbone. This region represents thermal decomposition rather than vitrification of the starch phase. The generally similar TGA and DTG profiles of the control, maltol-containing, and ethyl maltol-containing extrudates indicated that incorporation of 3% maltol or ethyl maltol did not markedly alter the overall thermal degradation behavior of the starch matrices.

The DSC thermograms of the starch extrudates are shown in Figure 3C. Most modified-starch samples exhibited broad endothermic events within approximately 250–350 °C, whereas the NT, NTE, and NTM samples showed different or less pronounced thermal profiles within this region. The onset of the major endothermic event occurred at approximately 230 °C. Because this temperature range overlapped with the substantial mass loss observed by TGA, the endothermic events were attributed primarily to thermal decomposition rather than to a reversible phase transition or melting of the starch materials. The modified starch extrudates generally began to undergo thermal degradation at lower temperatures than the native-starch samples, for which the principal degradation event occurred at approximately 300–320 °C [19,21]. This difference may be related to disruption of starch-chain organization by the substituent groups, the presence of glycerol, and interactions with the incorporated maltol derivatives.

Although maltol and ethyl maltol may affect molecular organization within the starch matrices, the high temperature DSC endotherms do not provide direct evidence that these compounds acted as nucleating agents or promoted crystallization. Such an interpretation would require supporting evidence from changes in relative crystallinity, crystallization exotherms, or controlled heating–cooling DSC measurements. Accordingly, the combined TGA and DSC results indicate that the principal high-temperature thermal events were dominated by decomposition and that the incorporation of maltol or ethyl maltol produced no substantial improvement or reduction in the overall thermal stability of the starch extrudates.

### 3.5. Mechanical Properties

The tensile properties of the starch extrudates, including tensile strength (TS), elongation at break (EB), and Young’s modulus (YM), are summarized in Table 2. Among the starch matrices examined, the OS, HH, and HL extrudates exhibited the highest TS, EB, and YM, respectively. These differences demonstrate that both the chemical nature of the starch substituent and, within the hydroxypropylated starch series, the substitution level influenced the mechanical behavior of the extrusion-plasticized materials.

The incorporation of 3% maltol or ethyl maltol generally increased the TS of the starch extrudates. Maltol and ethyl maltol are low-molecular-weight hydroxypyrone compounds containing hydroxyl and carbonyl groups capable of forming non-covalent interactions, particularly hydrogen bonds, with the hydroxyl groups of starch [22]. Their incorporation may therefore have altered starch-chain association and improved stress transfer within the extruded matrices. The increases in TS were consistent with the FTIR and NMR results, which indicated changes in the molecular environment of the starch following the addition of maltol or ethyl maltol. However, these compounds should not be described simply as promoting hydrogen bonding between starch chains because they may also compete with the original starch–starch hydrogen bonds and form new starch–compound associations.

The incorporation of maltol or ethyl maltol also generally increased the YM of the starch extrudates, indicating the formation of a stiffer matrix. This effect may have resulted from additional intermolecular associations that restricted starch-chain mobility under tensile loading. The relatively smooth external surfaces observed by SEM in several maltol- and ethyl maltol-containing extrudates were also consistent with improved matrix continuity. Nevertheless, irregular domains remained visible within some cross-sections. Therefore, the mechanical response was likely governed by a balance between molecular-level starch–compound interactions and the degree of structural heterogeneity within each starch matrix.

The effects of maltol and ethyl maltol on EB depended on the modified starch matrix. The addition of either compound increased EB in the HM, HL, and AC formulations, indicating improved extensibility. In these matrices, maltol and ethyl maltol may have increased local chain mobility while maintaining sufficient intermolecular interactions to preserve matrix integrity. Consequently, the materials were able to undergo greater deformation before fracture. The simultaneous improvement in TS and EB observed in some formulations suggests a favorable balance between intermolecular cohesion and chain mobility rather than a simple plasticizing or reinforcing effect.

In contrast, the incorporation of maltol or ethyl maltol reduced EB in the HH, NT, and OS formulations. The lower extensibility of these samples may have resulted from stronger starch–compound associations that restricted chain mobility or from structural heterogeneities that acted as stress-concentration sites and initiated fracture. For HH, the high hydroxypropyl substitution level may have altered the balance among starch–starch, starch–plasticizer, and starch–maltol derivative interactions. For OS, the amphiphilic octenyl succinate groups may have affected compatibility and the distribution of the incorporated compounds within the matrix. These matrix-dependent responses demonstrate that maltol and ethyl maltol did not function exclusively as plasticizers or reinforcing agents. Instead, their effects on mechanical performance depended on the chemistry and molecular organization of the modified starch matrix.

The results indicate that the mechanical properties of the extrudates were governed by the combined effects of starch modification chemistry, hydroxypropyl substitution level, and the incorporation of maltol or ethyl maltol. The OS formulation provided the greatest resistance to tensile failure, the HH formulation exhibited the greatest extensibility, and the HL formulation formed the stiffest matrix. The effects of the maltol derivatives were formulation-dependent, confirming that selection of the modified starch matrix is important for controlling the balance among strength, stiffness, and flexibility in extrusion-plasticized edible materials.

### 3.6. Antioxidant Activity of the Starch Extrudates

The release of DPPH radical-scavenging activity from the starch extrudates is depicted in Figure 4, demonstrating the antioxidant activity of the incorporated maltol and ethyl maltol. DPPH is a stable free radical because its unpaired electron is delocalized over the molecule, while the aromatic structure and steric effects provide additional stability. Maltol and ethyl maltol can donate hydrogen atoms or electrons to the DPPH radical, thereby reducing the absorbance of the solution. The resulting decrease in absorbance was used to quantify the free radical-scavenging activity of the samples.

Although the different starch types showed varying antioxidant capacities, maltol and ethyl maltol exhibited comparable DPPH radical-scavenging activities [6,23]. Hydroxypropylated starch with a low substitution grade (HL) and octenyl succinate-modified starch (OS) showed relatively high DPPH radical-scavenging responses, indicating greater accessibility of antioxidant-active compounds to the assay medium under the conditions used. Because the concentrations of maltol and ethyl maltol in the surrounding medium were not directly quantified, these results should not be interpreted as evidence of greater chemical release. Furthermore, NT showed lower accessible radical-scavenging activity than several modified starch formulations, including HH, HM, HL, OS, and AC. The bulky hydroxypropyl, octenyl succinate, and acetyl groups may have prevented close packing of the starch chains and facilitated greater penetration of the release medium into the starch extrudate matrices. This greater medium accessibility may have increased the contact between the assay solution and antioxidant-active compounds within the starch matrices, thereby increasing the measured DPPH radical-scavenging response.

### 3.7. Antimicrobial Activities of the Starch Extrudates

#### 3.7.1. Antibacterial Activities

Figure 5A shows the results of the disk diffusion method used to assess the antibacterial activity of the starch extrudates against *B. subtilis*, *S. aureus*, and *E. coli*. After 24 h of incubation, the control without a starch extrudate showed no inhibition of bacterial growth. No inhibition zones against *S. aureus* or *E. coli* were observed around the starch extrudates. Therefore, antibacterial activity against these two bacteria could not be demonstrated by the disk diffusion method under the conditions used [7,8,9].

In contrast, the HME, HLM, and HHE samples produced inhibition zones against *B. subtilis* of 2.52, 2.24, and 3.53 mm, respectively. Among these samples, HHE produced the largest inhibition zone. Differences in antibacterial activity may be associated with the molecular organization of the starch matrices, which could affect the dispersion, accessibility, and diffusion of the antimicrobial compounds. Greater starch-chain packing or crystallization may restrict the diffusion of maltol and ethyl maltol from the extrudates into the agar medium, thereby reducing the observed inhibition zones.

Maltol and ethyl maltol showed limited antibacterial activity after incorporation into the starch matrices, with no clear inhibition of *S. aureus* or *E. coli*. However, selected hydroxypropylated starch extrudates containing maltol or ethyl maltol showed measurable inhibitory effects against *B. subtilis*. These findings indicate that the antibacterial activity of the extrudates depended on both the bacterial species and the modified starch matrix.

#### 3.7.2. Antifungal Activities

Figure 5B–D show the antifungal activity of the starch extrudates against *Aspergillus niger*, *Penicillium* sp., and *Rhizopus* sp., as determined using the disk diffusion method. The HLE, HLM, NTE, NTM, OSE, ACE, and ACM extrudates produced the largest inhibition zones against *A. niger*, with diameters of 6.54, 4.13, 6.09, 4.90, 7.39, 5.99, and 4.22 mm, respectively, as shown in Figure 5B and Table 3. Inhibition zones were observed around the margins of the starch extrudates on the first day of incubation. Growth-free areas also developed in other regions of the agar medium. These observations suggest that maltol and ethyl maltol may have been released from the extrudates, migrated through the headspace, and subsequently been absorbed into the agar medium, resulting in antifungal activity beyond the regions of direct contact. However, confirmation of vapor-phase release would require a separate experimental arrangement that prevents direct diffusion through the agar.

The development of a dark brown coloration on the surface of the medium indicated conidial formation by *A. niger* during incubation for up to five days. No comparable brown coloration was observed in the areas associated with the starch extrudates containing maltol or ethyl maltol, indicating delayed conidial development and sporulation of *A. niger* [7,24]. After five days of incubation, the mold colonies changed from white to dark brown as sporulation progressed. Differences in the distribution of the growth-free areas also suggested possible differences between the release or migration behavior of maltol and ethyl maltol.

The antifungal activity of the starch extrudates against *Penicillium* sp. is shown in Figure 5C. On day 1 of incubation, clear zones without visible fungal growth were observed around the extrudates (Table 3). This observation indicates that maltol and ethyl maltol were released from the starch extrudates and diffused into the surrounding agar medium. Inhibition zones were observed for the HME, HMM, HLE, HLM, NTE, NTM, OSE, OSM, ACE, and ACM extrudates, with measured diameters of 0.34, 0.55, 0.18, 0.52, 0.35, 0.35, 0.31, 0.34, 0.32, and 0.42 mm, respectively. The inhibition zones remained visible after five days of incubation, demonstrating persistent antifungal activity associated with direct contact and the continued diffusion of maltol or ethyl maltol from the extrudates into the surrounding medium.

The antifungal activity of the starch extrudates against *Rhizopus* sp. is shown in Figure 5D. No distinct inhibition zones were observed around the extrudates containing maltol or ethyl maltol. After five days of incubation, fungal growth and conidial development were visible on the surface of the medium, as indicated by the formation of *Rhizopus* sp. spores. Although no clear inhibition zones were detected, maltol appeared to delay sporulation more effectively than ethyl maltol. This difference was observed despite the apparently greater release of ethyl maltol, suggesting that the antifungal response was not determined solely by the amount released. Differences in the intrinsic antifungal activity, availability, or interaction of each compound with the fungal cells may also have contributed to the observed response.

### 3.8. Integrated Structure–Interaction–Property Relationships

The results collectively indicate that the behavior of maltol and ethyl maltol in extrusion-plasticized starch matrices was governed not by the presence of the flavor compounds alone, but by the molecular environment created by each starch modification. Acetyl, octenyl succinate, and hydroxypropyl substituents altered starch-chain association, polarity, and packing, thereby changing the ability of the matrix to accommodate and interact with the incorporated compounds. Within the hydroxypropylated starch series, increasing substitution reduced the regularity of starch-chain packing, as reflected by the progressive decrease in diffraction intensity. This suggests that hydroxypropyl substitution introduced steric disruption into the starch network, which increased structural disorder but did not produce a uniform effect on mechanical performance. Instead, the resulting behavior depended on the balance between enhanced chain mobility and the loss of regular intermolecular packing.

The distinction between maltol and ethyl maltol further demonstrates that compound–matrix interactions were influenced by the molecular structure of the incorporated flavor compound. Maltol-containing extrudates retained a distinct diffraction peak at approximately 2θ = 14.7°, indicating the presence of residual or recrystallized maltol domains, whereas no comparable crystalline peak was detected for ethyl maltol. This difference suggests that ethyl maltol remained in a less ordered or more highly dispersed state within the starch matrices. In combination with the FTIR and NMR results, these observations support the view that the two compounds experienced different local molecular environments after extrusion. However, because the present analyses cannot distinguish unequivocally among hydrogen bonding, physical entrapment, and other non-covalent associations, these interactions are interpreted as matrix-dependent molecular associations rather than as formation of a specific chemical bond.

These molecular and structural differences provide a basis for understanding why maltol and ethyl maltol did not act simply as either plasticizers or reinforcing agents. Their mechanical effects were strongly formulation-dependent. In some matrices, incorporation of the maltol derivatives increased both tensile strength and extensibility, suggesting that additional intermolecular associations were formed without excessively restricting chain motion. In other formulations, increased stiffness was accompanied by reduced elongation, indicating that stronger local association or greater structural heterogeneity limited chain mobility during deformation. Thus, the mechanical response resulted from a balance among starch–starch, starch–glycerol, and starch–maltol derivative interactions rather than from a single reinforcing or plasticizing mechanism.

The same matrix dependence was evident in the functional responses. Native starch showed relatively low release-associated antioxidant activity, whereas several modified starch matrices, particularly hydroxypropylated and octenyl succinate-modified starches, showed greater DPPH radical-scavenging responses. This behavior is consistent with the idea that bulky substituent groups disrupted dense starch-chain packing and increased accessibility of the release medium to the incorporated compounds. Nevertheless, greater compound accessibility did not translate directly into stronger antimicrobial activity in all cases. Antibacterial effects were limited mainly to selected hydroxypropylated formulations against *B. subtilis*, while antifungal activity varied substantially among starch matrices and fungal species. These findings indicate that functional performance depended not only on compound release but also on the susceptibility of the target microorganism and the way in which each compound was retained and transported through the starch matrix.

Taken together, the central finding is that the modified starch should not be regarded as an inert carrier for maltol or ethyl maltol. Rather, its substitution chemistry and level of modification determined the molecular environment of the incorporated compounds and consequently influenced their physical state, chain interactions, mechanical response, and functional availability. The results therefore support the original hypothesis that starch modification chemistry and hydroxypropyl substitution level regulate the structure–interaction–property relationships of extrusion-plasticized edible starch materials. Importantly, the relationship was not monotonic: increasing substitution did not uniformly improve all properties, but instead shifted the balance among molecular packing, mobility, compound retention, and release. This matrix-specific behavior should therefore be considered when selecting modified starches for edible systems requiring a particular combination of flexibility, structural integrity, flavor functionality, antioxidant activity, and selective antimicrobial performance.

## 4. Conclusions

Native and modified cassava starches containing either 3% ethyl maltol or 3% maltol were successfully processed by twin-screw extrusion, resulting in structural and morphological changes that influenced their mechanical, antioxidant, and antimicrobial properties. The modified starches included acetylated and octenyl succinate-modified starches, together with hydroxypropylated starches with low, medium, and high substitution levels. Changes in the FTIR and NMR spectra indicated that incorporation of maltol or ethyl maltol altered the molecular environment of the starch matrices. These spectral changes were consistent with possible non-covalent associations among starch, glycerol, and the maltol derivatives; however, the present spectroscopic data do not establish a specific interaction mechanism. The incorporation of maltol and ethyl maltol also altered the tensile strength, Young’s modulus, and elongation at break of the extrudates, with the magnitude and direction of these changes depending on the starch matrix. XRD analysis showed that the extrudates retained different degrees of ordered or semicrystalline structure. The more distinct diffraction peaks in the maltol-containing extrudates suggested the presence of residual or recrystallized maltol domains, whereas the absence of corresponding ethyl maltol peaks indicated a less ordered or more highly dispersed state. The starch extrudates released compounds with DPPH radical-scavenging activity and exhibited matrix- and microorganism-dependent antimicrobial effects. Selected hydroxypropylated starch formulations inhibited *Bacillus subtilis*, whereas no distinct inhibition zones against *Staphylococcus aureus* or *Escherichia coli* were observed under the conditions tested. The extrudates also inhibited *Aspergillus niger* and *Penicillium* sp., while maltol delayed the sporulation of *Rhizopus* sp. without producing a distinct inhibition zone. The results demonstrate that modified cassava starch chemistry and hydroxypropyl substitution level influenced the interactions, structure, and functional performance of extrusion-plasticized starch materials containing maltol or ethyl maltol. These materials therefore show potential as flexible edible starch matrices containing maltol or ethyl maltol, with measurable antioxidant activity and selective, microorganism-dependent antimicrobial effects. Further studies using actual food systems are required to determine their effects on sensory quality, microbial stability, shelf life, and food-waste reduction. The principal advantage of this approach is that a continuous extrusion process can generate flexible edible starch matrices in which structural performance and selected antioxidant and antimicrobial functions can be modulated through starch modification chemistry and substitution level. The findings therefore provide a basis for selecting modified starch matrices according to the desired balance of mechanical integrity, flexibility, and functional performance. However, further optimization is required because only one maltol/ethyl maltol concentration and one extrusion condition were evaluated. Future studies should quantify compound retention and release kinetics, optimize formulation and processing variables, and validate sensory quality, microbial stability, and shelf-life performance in actual food systems.

## Figures and Tables

**Figure 1:**
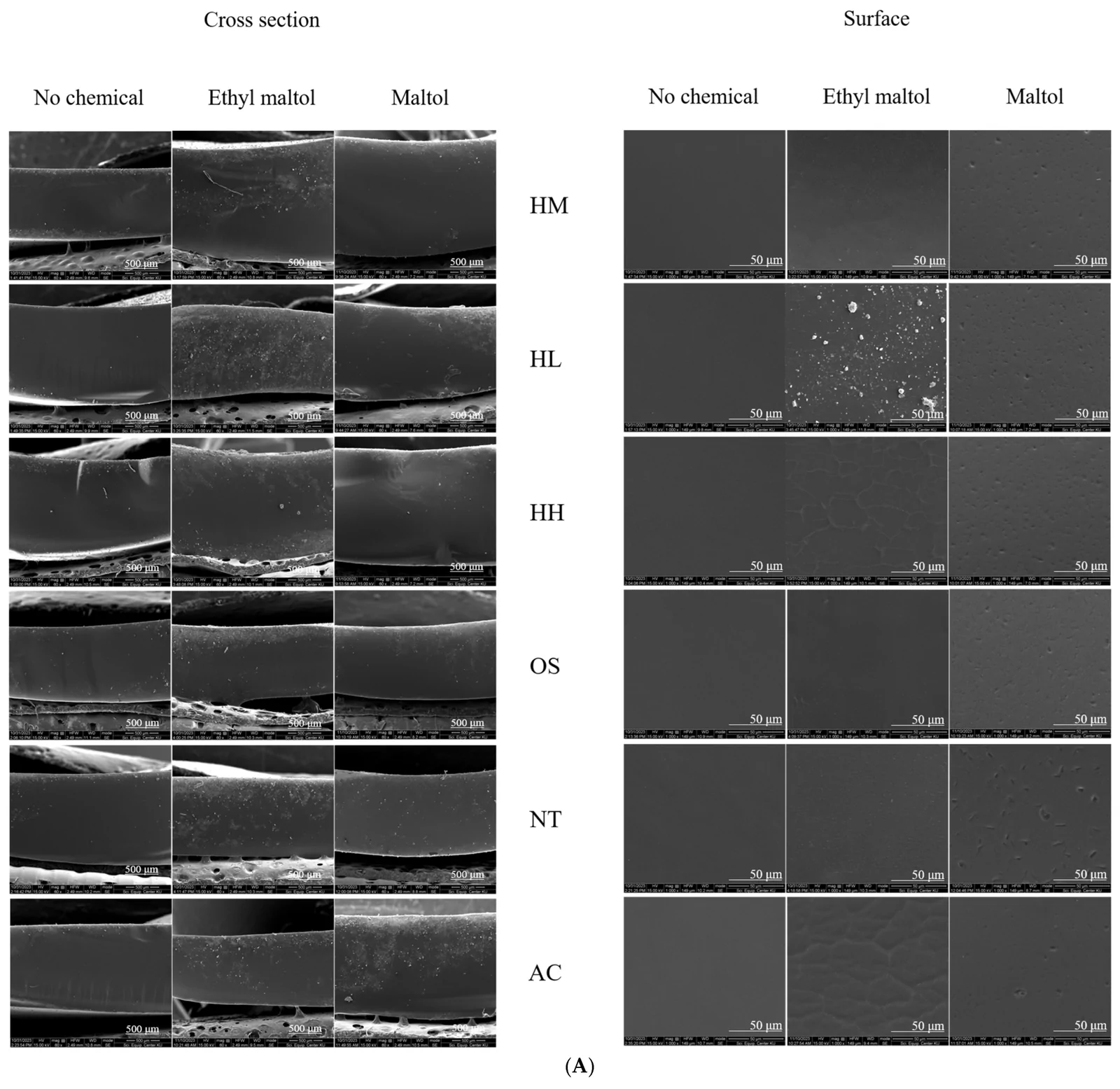

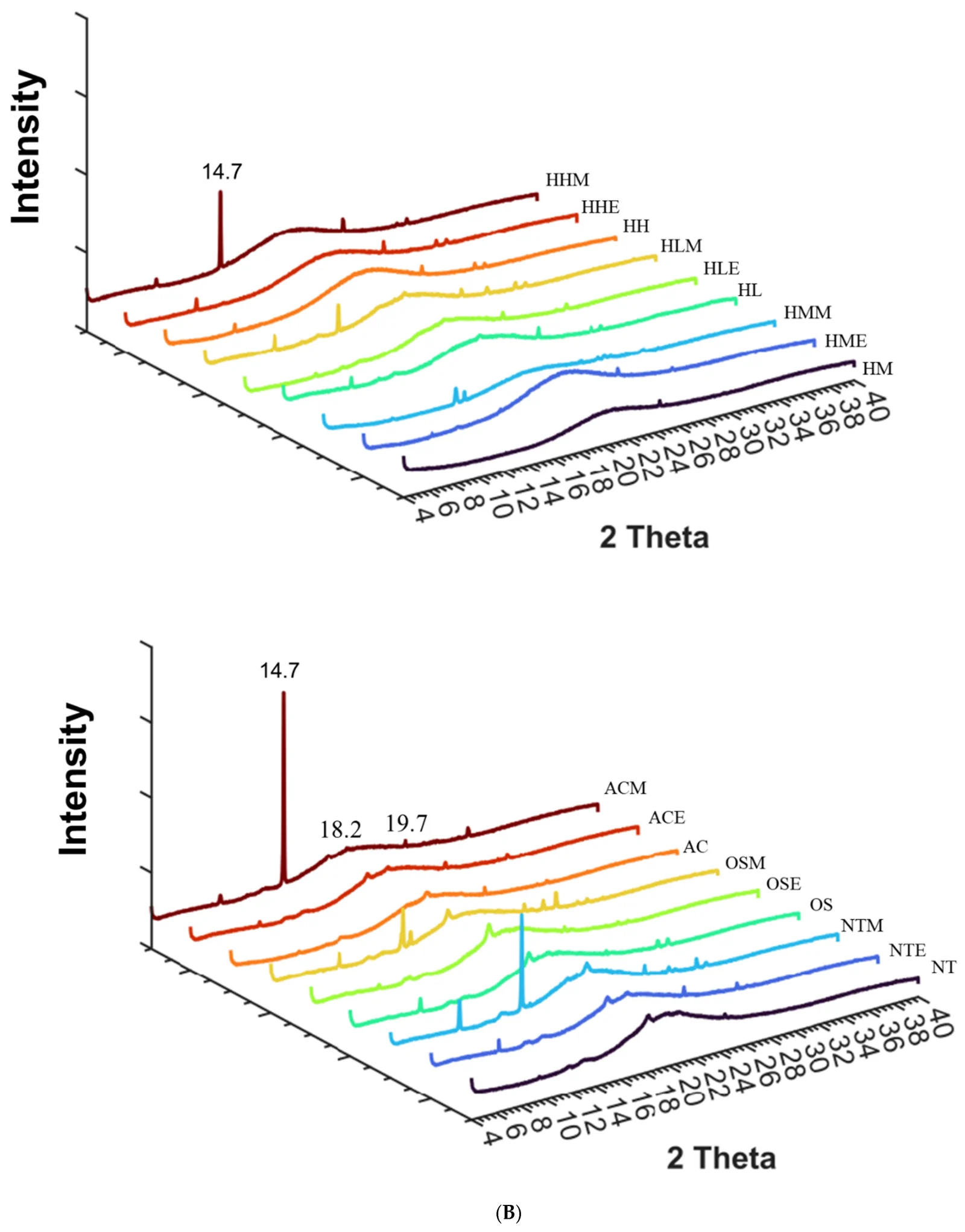
(**A**) SEM micrographs of the cross-sections and surfaces and (**B**) X-ray diffraction (XRD) patterns of starch extrudates prepared from native starch (NT), acetylated starch (AC), octenyl succinate-modified starch (OS), and hydroxypropylated starch with low (HL), medium (HM), and high (HH) substitution levels, without or with 3% ethyl maltol (E) or maltol (M).

**Figure 2:**
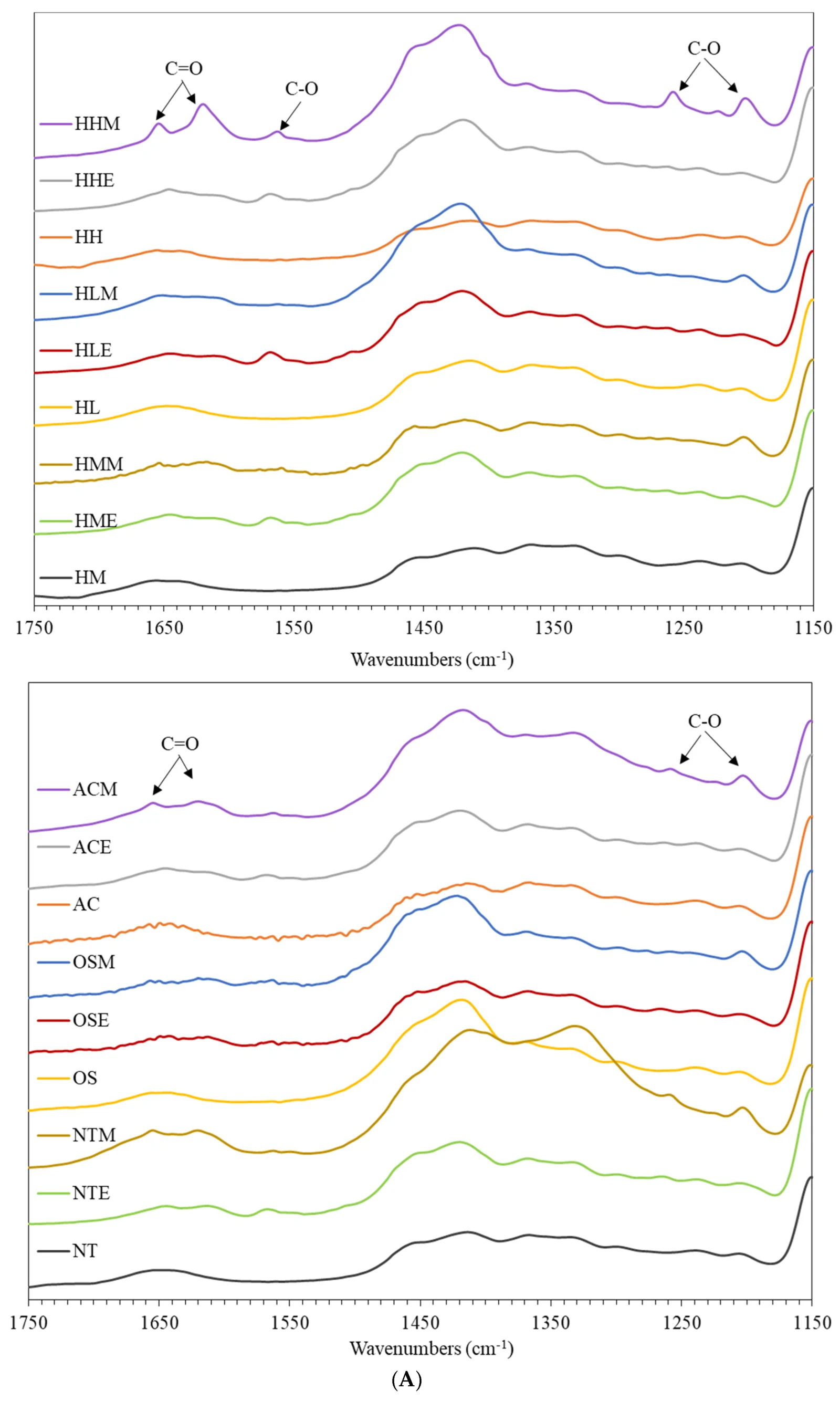

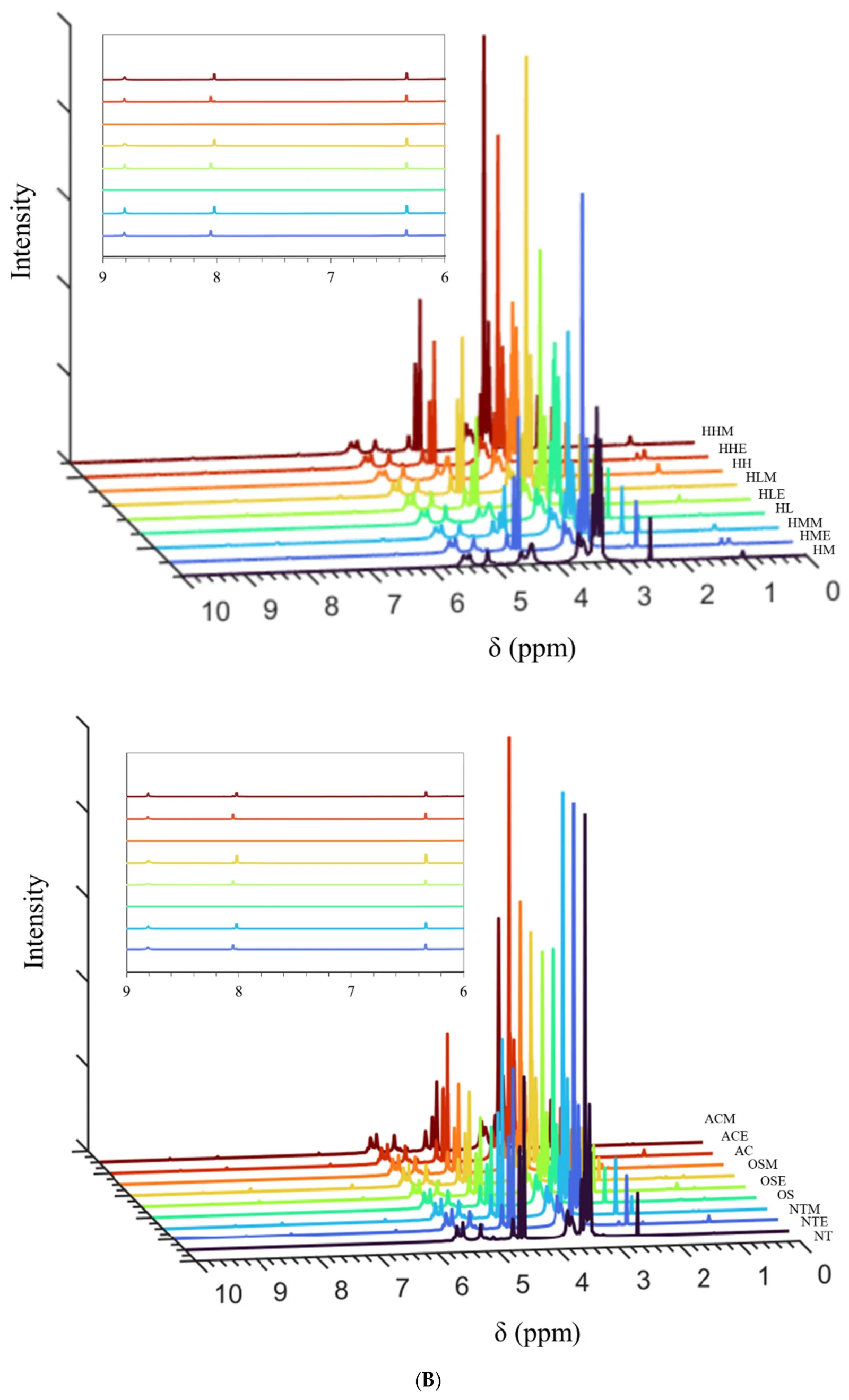
(**A**) ATR-FTIR spectra and (**B**) ^1^H NMR spectra of starch extrudates prepared from native starch (NT), acetylated starch (AC), octenyl succinate-modified starch (OS), and hydroxypropylated starch with low (HL), medium (HM), and high (HH) substitution levels, without or with 3% ethyl maltol (E) or maltol (M).

**Figure 3:**
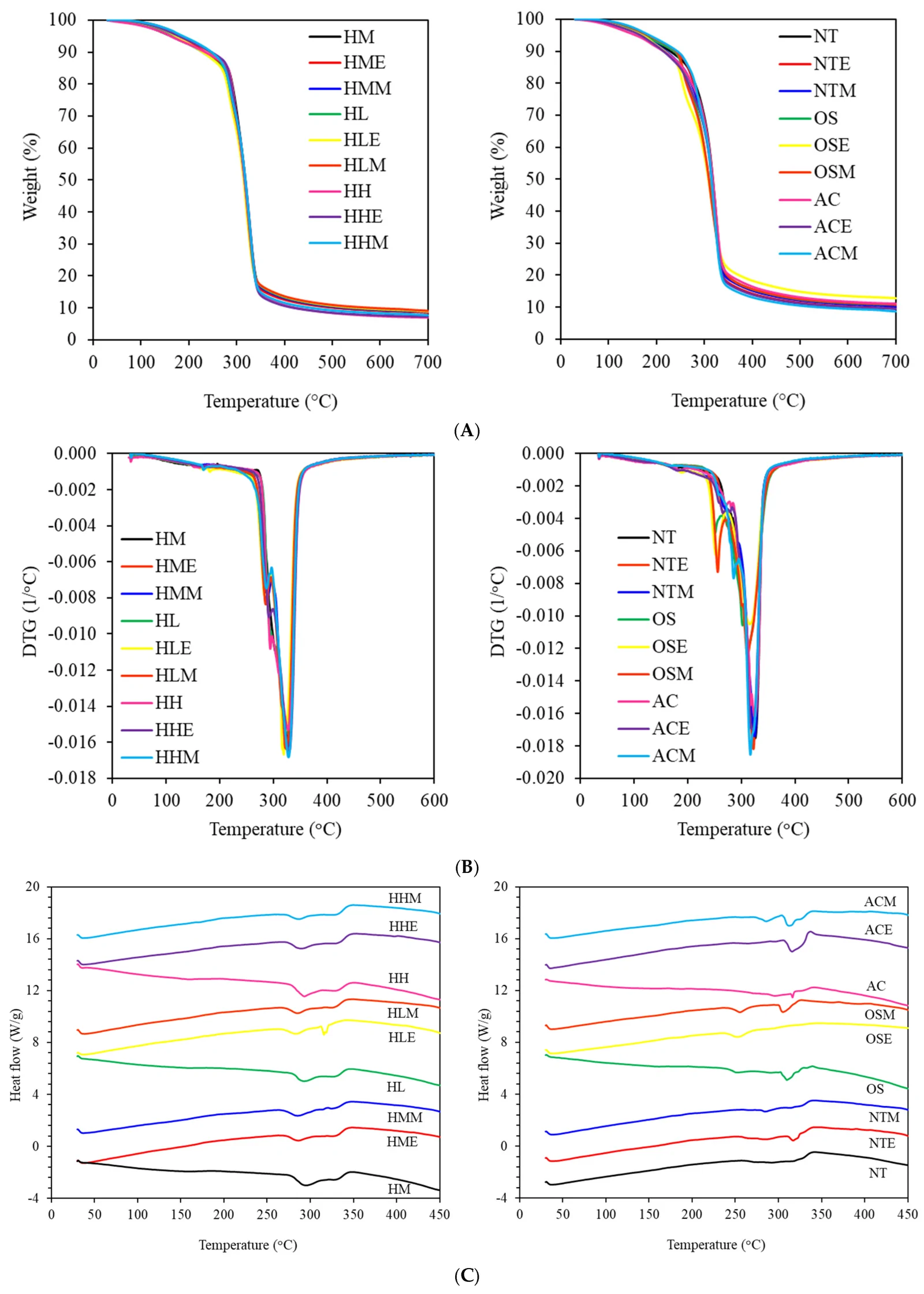
Thermal degradation behavior of starch extrudates (native starch (NT), acetylated starch (AC), octenyl succinate (OS), hydroxypropylated low DS (HL), hydroxypropylated medium DS (HM), and hydroxypropylated high DS (HH)) with ethyl maltol (E) and maltol (M) at 3%, as (**A**) weight loss, (**B**) first derivative weight loss, and (**C**) DSC thermograms.

**Figure 4:**
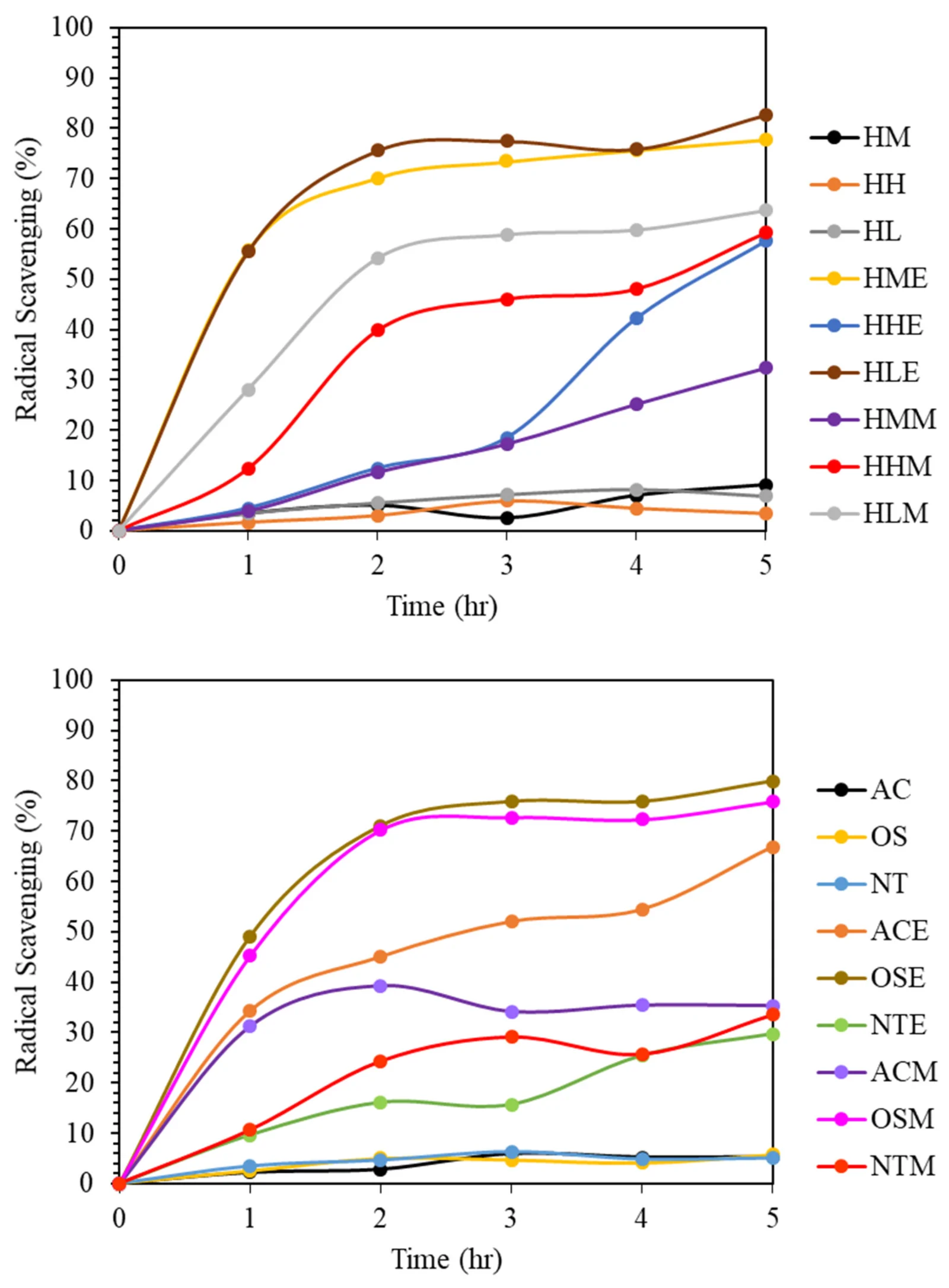
DPPH radical-scavenging activity measured for starch extrudates prepared from native starch (NT), acetylated starch (AC), octenyl succinate-modified starch (OS), and hydroxypropylated starch with low (HL), medium (HM), and high (HH) supplier-defined substitution grades, containing 3% ethyl maltol (E) or maltol (M), as a function of assay time.

**Figure 5:**
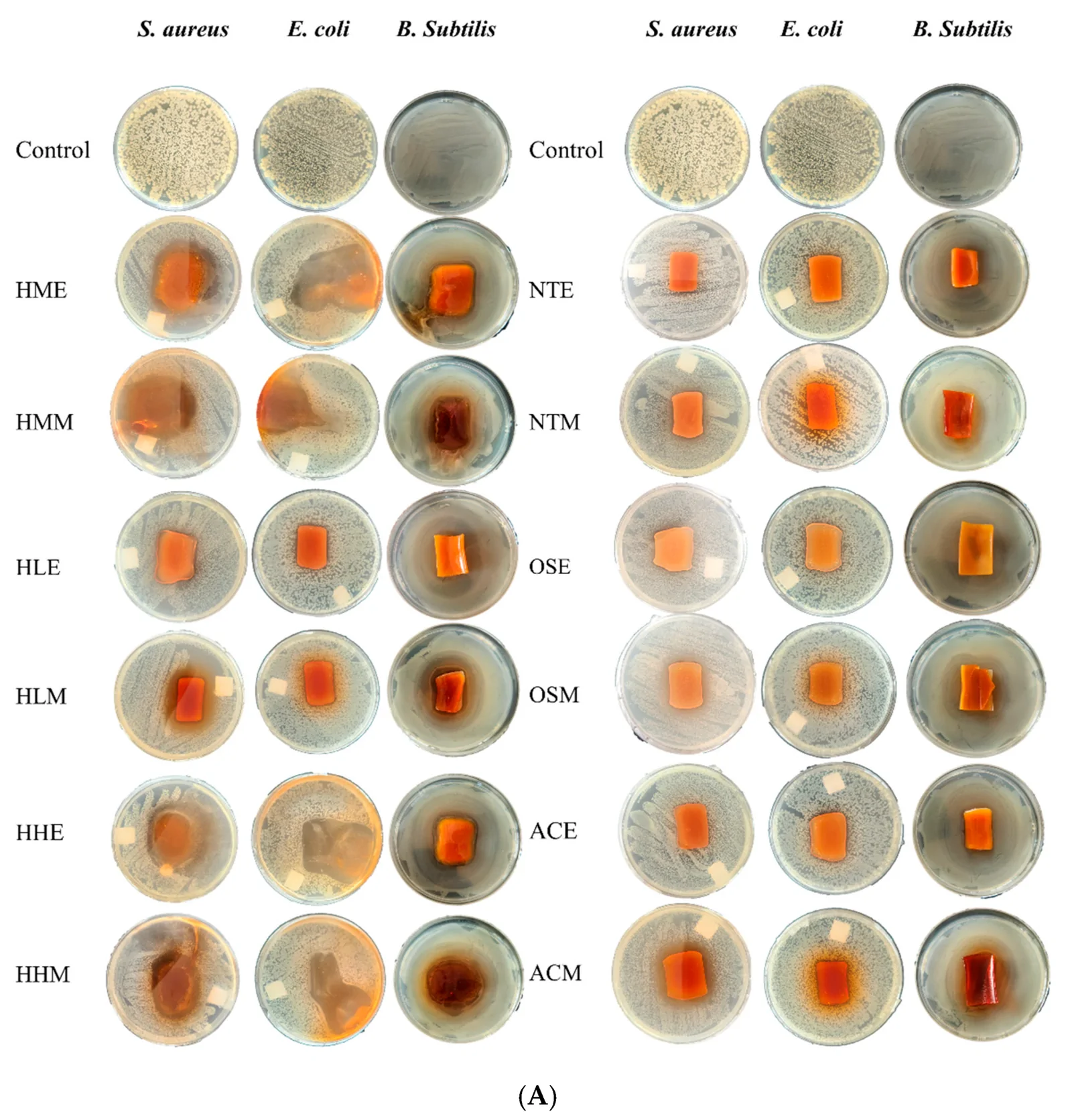

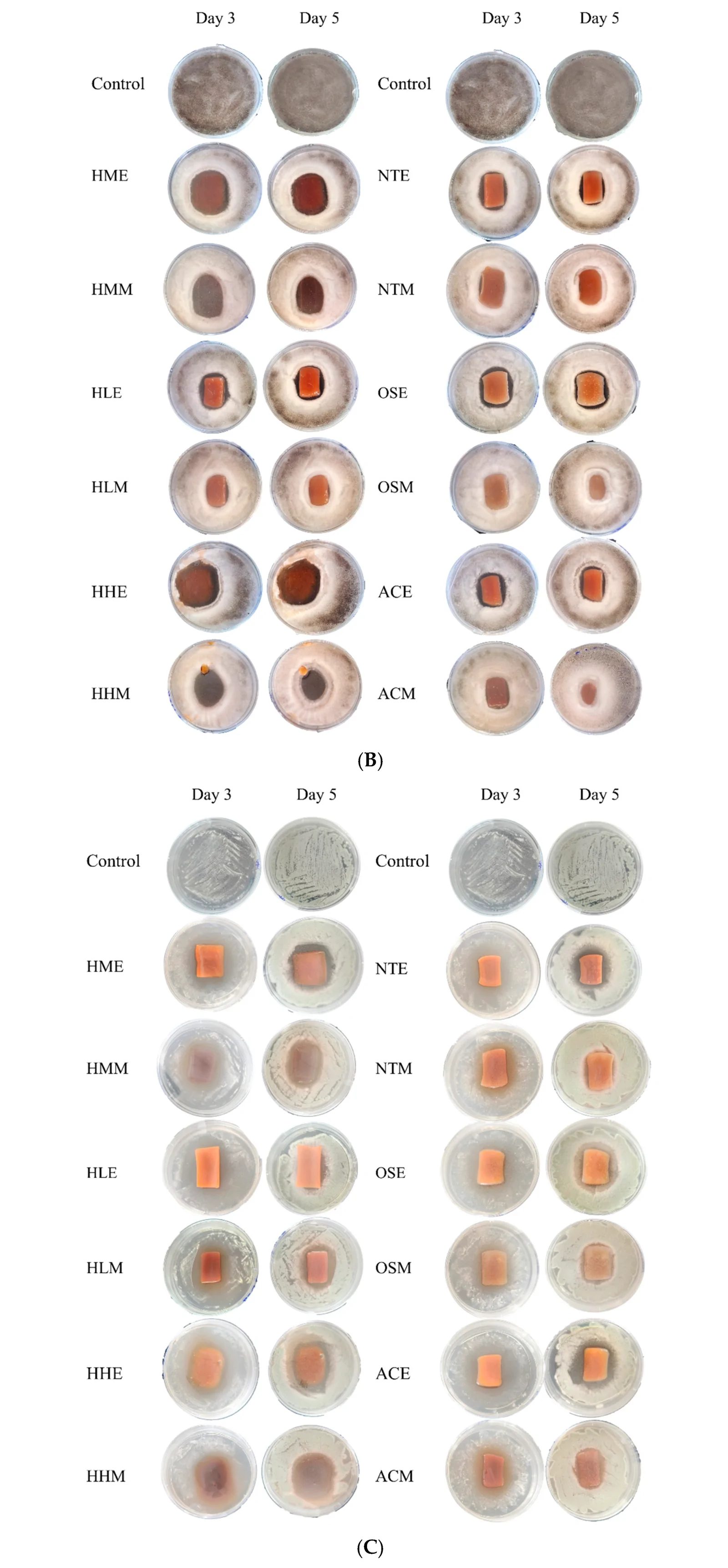

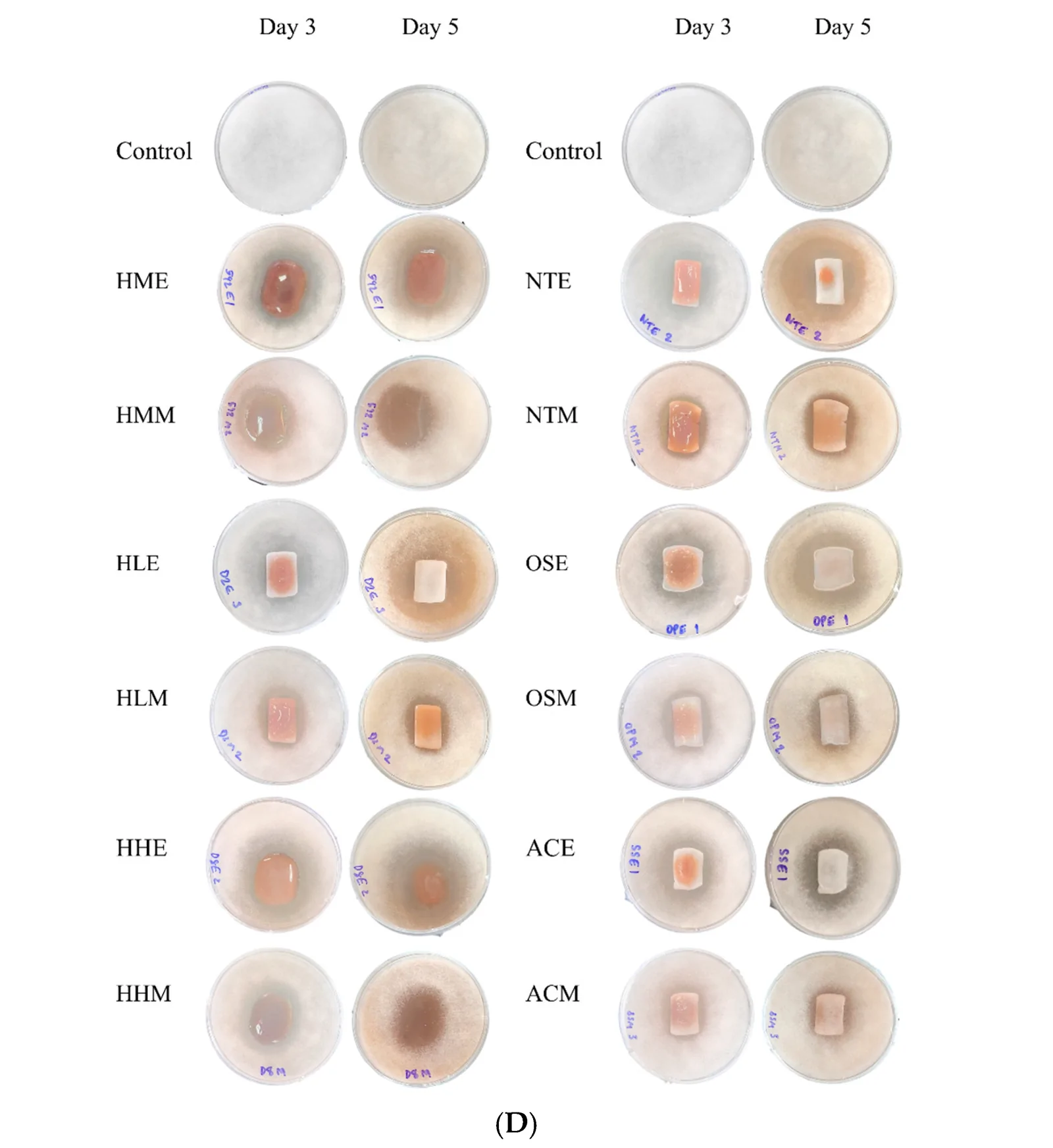
In vitro antibacterial performance against *Staphylococcus aureus*, *Escherichia coli*, and *Bacillus subtilis* (**A**) and antifungal performance against *Aspergillus niger* (**B**), *Penicillium* sp. (**C**) and *Rhizopus* sp. (**D**) of starch extrudates (native starch (NT), acetylated starch (AC), octenyl succinate (OS), hydroxypropylated low DS (HL), hydroxypropylated medium DS (HM), and hydroxypropylated high DS (HH)) containing 3% of ethyl maltol (E) and maltol (M).

**Table 1:** Formulations of starch extrudates with ethyl maltol (E) and maltol (M).

Starch		Ethyl Maltol	Maltol
Neat starch	NT	NTE	NTM
Acetylated starch low DS (0.01–0.03)	AC	ACE	ACM
Octenyl succinate (DS: 0.01–0.03)	OS	OSE	OSM
Hydroxypropylated low DS (<0.07)	HL	HLE	HLM
Hydroxypropylated medium DS (0.07–0.09)	HM	HME	HMM
Hydroxypropylated high DS (>0.09)	HH	HHE	HHM

**Table 2:** Tensile strength (TS), elongation at break (EB) and Young’s Modulus (YM) of starch extrudates.

Starch Sheet	TS (MPa)	EB (%)	YM (MPa)
HM	0.30 ± 0.05 ^k^	425.00 ± 36.10 ^c^	0.51 ± 0.05 ^h^
HME	0.33 ± 0.02 ^k^	471.00 ± 9.00 ^b^	0.33 ± 0.07 ^h^
HMM	2.10 ± 0.07 ^de^	225.00 ± 8.70 ^defg^	33.30 ± 4.50 ^de^
HL	0.91 ± 0.04 ^i^	167.00 ± 9.30 ^g^	7.26 ± 0.44 ^gh^
HLE	1.86 ± 0.02 ^f^	255.00 ± 11.30 ^de^	16.60 ± 0.70 ^ef^
HLM	2.34 ± 0.03 ^cd^	218.00 ± 4.80 ^efg^	46.00 ± 4.00 ^bc^
HH	0.18 ± 0.01 ^k^	552.00 ± 44.20 ^a^	0.22 ± 0.04 ^h^
HHE	3.56 ± 0.08 ^a^	268.00 ± 10.30 ^d^	108.00 ± 7.70 ^a^
HHM	2.81 ± 0.07 ^b^	242.00 ± 4.10 ^defg^	53.80 ± 0.46 ^b^
NT	0.60 ± 0.03 ^i^	249.00 ± 13.40 ^def^	1.38 ± 0.14 ^h^
NTE	2.59 ± 0.07 ^bc^	222.00 ± 3.70 ^efg^	43.80 ± 9.50 ^c^
NTM	1.79 ± 0.08 ^g^	227.00 ± 15.50 ^def^	4.41 ± 0.58 ^h^
OS	1.59 ± 0.11 ^h^	226.00 ± 8.40 ^defg^	6.19 ± 1.19 ^gh^
OSE	2.65 ± 0.03 ^b^	193.00 ± 4.34 ^fg^	44.70 ± 1.63 ^c^
OSM	2.85 ± 0.05 ^b^	197.00 ± 3.66 ^fg^	47.20 ± 1.08 ^bc^
AC	0.43 ± 0.05 ^i^	186.00 ± 8.82 ^fg^	3.81 ± 0.43 ^h^
ACE	3.50 ± 0.11 ^a^	268.00 ± 10.27 ^de^	108.00 ± 7.66 ^a^
ACM	2.17 ± 0.13 ^fg^	255.00 ± 7.09 ^def^	14.44 ± 2.22 ^fg^

NT: native starch, AC: acetylated starch, OS: octenyl succinate, HL: hydroxypropylated low DS, HM: hydroxypropylated medium DS, HH: hydroxypropylated high DS with ethyl maltol (E) and maltol (M) at 3%. The values are represented as a mean ± standard deviation. In the same column, any two means, followed by the same letter, are not significantly different (*p* > 0.05).

**Table 3:** Inhibition zones of starch extrudates against the growth of *Bacillus subtilis*, *Aspergillus niger*, and *Penicillium* sp.

	Inhibition Zone (mm)		
Starch Extrudates	*Bacillus subtilis*	*Aspergillus niger*	*Penicillium* sp.
HME	2.52 ± 0.03	-	0.34 ± 0.01
HMM	-	-	0.55 ± 0.03
HLE	-	6.54 ± 0.74	0.18 ± 0.02
HLM	2.24 ± 0.51	4.13 ± 0.24	0.52 ± 0.06
HHE	3.53 ± 0.28	-	-
HHM	-	-	-
NTE	-	6.09 ± 0.73	0.35 ± 0.05
NTM	-	4.90 ± 1.02	0.35 ± 0.05
OSE	-	7.39 ± 0.44	0.31 ± 0.04
OSM	-	-	0.34 ± 0.14
ACE	-	5.99 ± 0.88	0.32 ± 0.04
ACM	-	4.22 ± 0.26	0.42 ± 0.02

NT: native starch, AC: acetylated starch, OS: octenyl succinate, HL: hydroxypropylated low DS, HM: hydroxypropylated medium DS, HH: hydroxypropylated high DS with ethyl maltol (E) and maltol (M) at 3%.

## Data Availability

The original contributions presented in this study are included in the article/Supplementary Materials. Further inquiries can be directed to the corresponding authors.

## Supplementary Materials

The following supporting information can be downloaded at: https://www.mdpi.com/article/10.3390/foods15193393/s1, Figure S1: Appearance and opacity (A600/Thickness (AU nm/mm) where A600 is the absorbance at 600 nm) of the starch extrudates (A), and light transmission (B) of starch extrudates (native starch (NT), acetylated starch (AC), octenyl succinate (OS), hydroxypropylated low DS (HL), hydroxypropylated medium DS (HM), and hydroxypropylated high DS (HH)) with ethyl maltol (E) and maltol (M) at 3%; Figure S2: Intensity of the NMR peak at 5.106 ppm (C) of starch extrudates (native starch (NT), acetylated starch (AC), octenyl succinate (OS), hydroxypropylated low DS (HL), hydroxypropylated medium DS (HM), and hydroxypropylated high DS (HH)) with ethyl maltol (E) and maltol (M) at 3%; Figure S3: Molecular weights of the native starch (NT), acetylated starch (AC), octenyl succinate (OS), hydroxypropylated low DS (HL), hydroxypropylated medium DS (HM), and hydroxypropylated high DS (HH) with ethyl maltol (E) and maltol (M) at 3% were determined by Matrix-Assisted Laser Desorption Ionization-Time of Flight Mass Spectrometry (MALDI-TOF MS) in reflectron mode.
