# Supplementary material for: Flexible Edible Materials from Extrusion-Plasticized Modified Cassava Starches with Maltol and Ethyl Maltol: Molecular Interactions, Mechanical Properties, and Antioxidant and Antimicrobial Activities
Source: Foods. 2026 Sep 23;15(19):3393. doi: 10.3390/foods15193393 (PMC13664071; doi:10.3390/foods15193393)
Supplement: Supplementary file 1 [file foods-15-03393-s001.zip › foods-4576888-supplementary.pdf]

Supplementary data

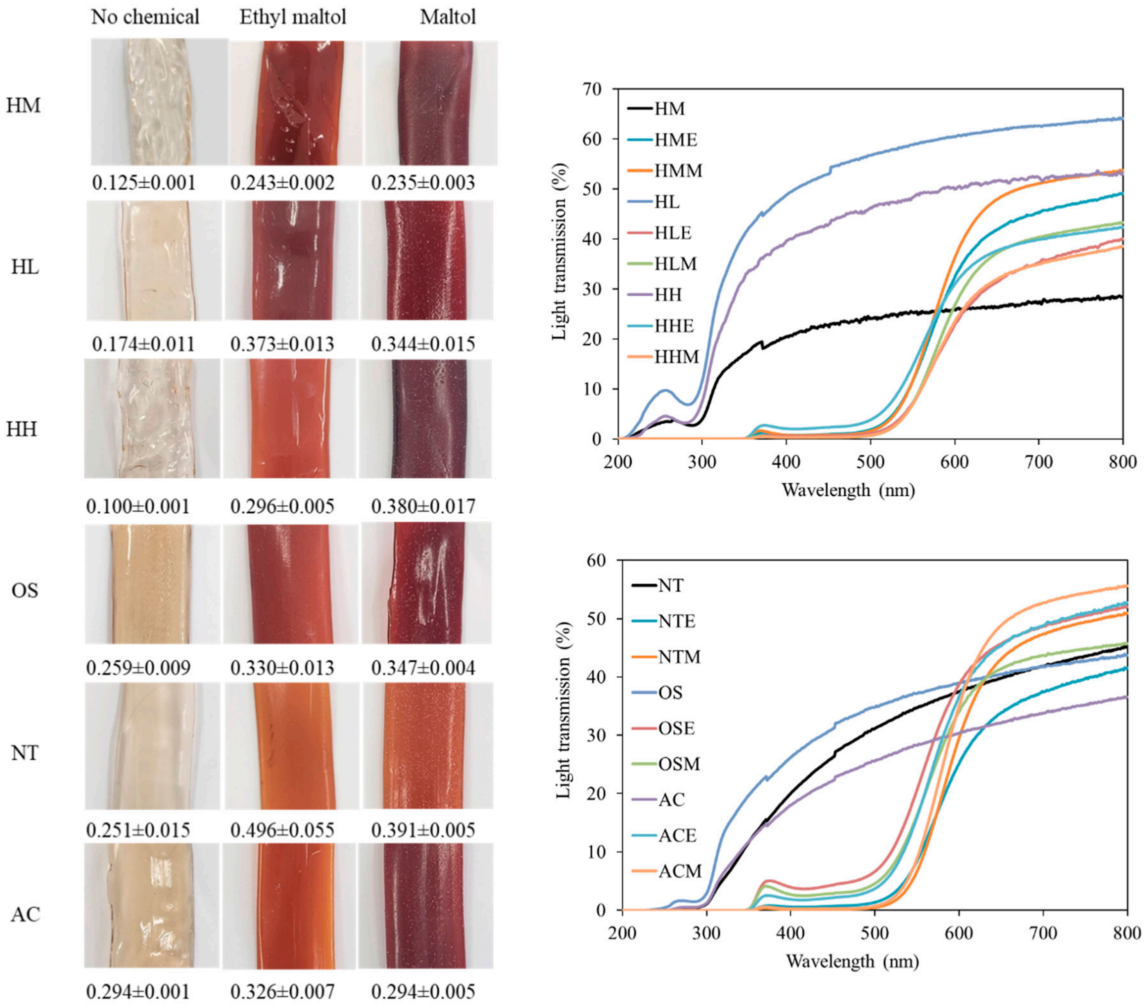

(A) (B)

Fig. S1. appearance and opacity (A_{600}/Thickness (AU nm/mm) where A_{600} is the absorbance at 600 nm) of the starch extrudates (A), and light transmission (B) of starch extrudates (native starch (NT), acetylated starch (AC), octenyl succinate (OS), hydroxypropylated low DS (HL), hydroxypropylated medium DS (HM), and hydroxypropylated high DS (HH)) with ethyl maltol (E) and maltol (M) at 3%

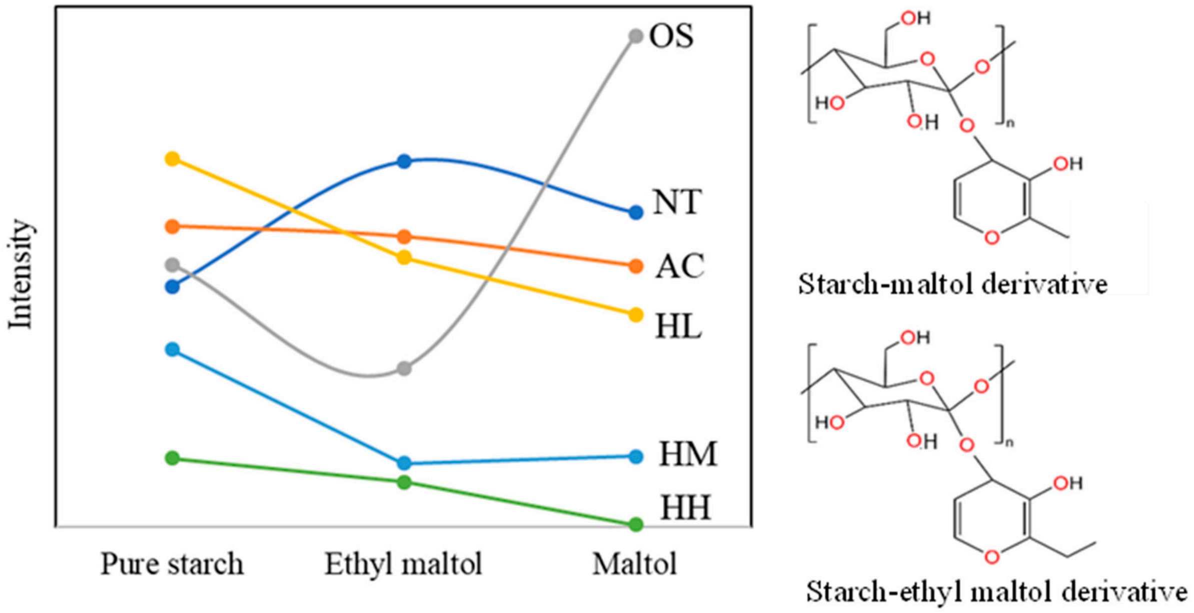

Fig. S2. intensity of the NMR peak at 5.106 ppm (C) of starch extrudates (native starch (NT), acetylated starch (AC), octenyl succinate (OS), hydroxypropylated low DS (HL), hydroxypropylated medium DS (HM), and hydroxypropylated high DS (HH)) with ethyl maltol (E) and maltol (M) at 3%.

Native starch (NT)

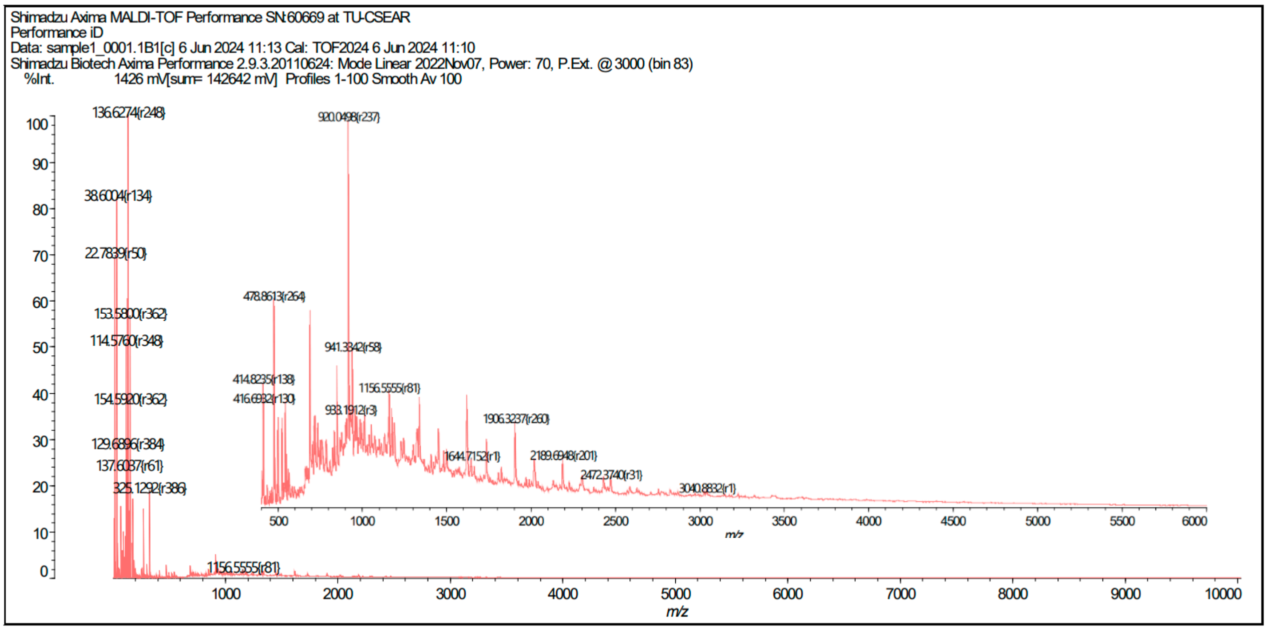

Octenyl succinated starch (OS)

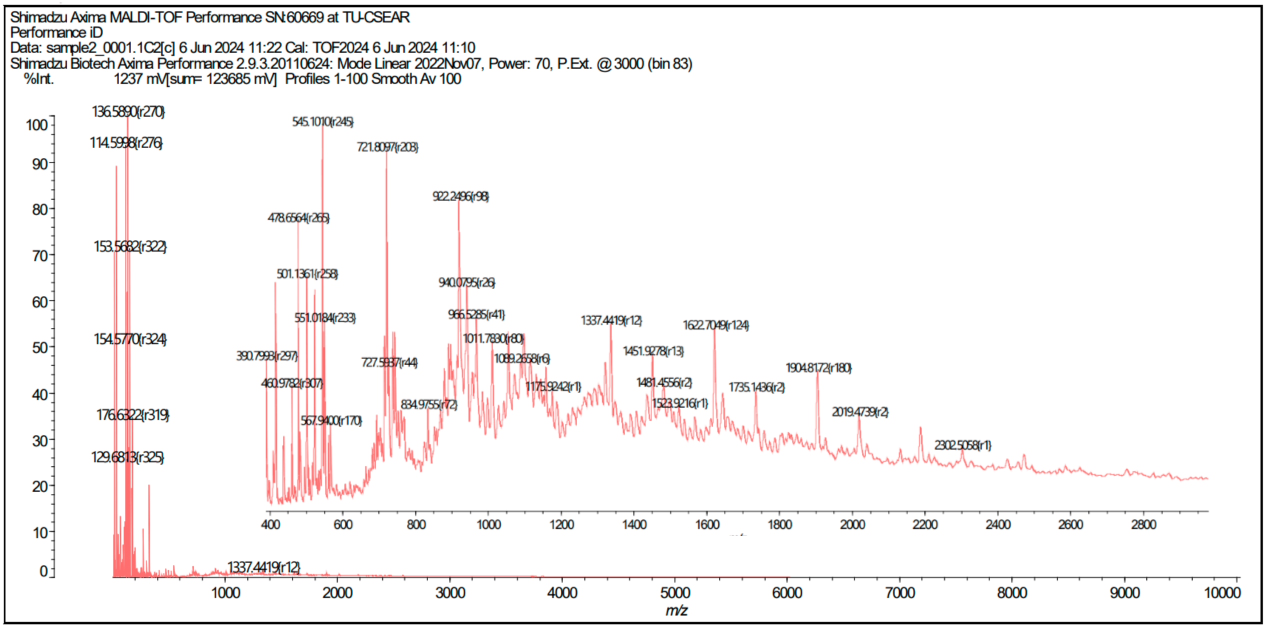

Acetylated starch (AC)

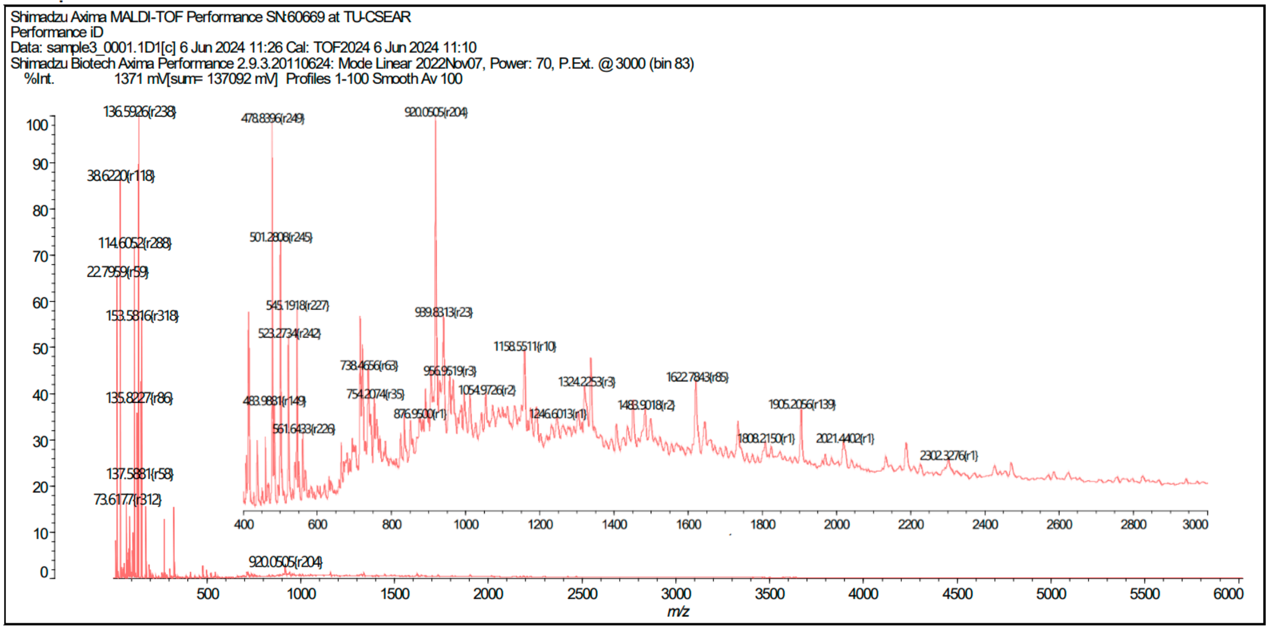

Hydroxypropylated starch medium DS (HM)

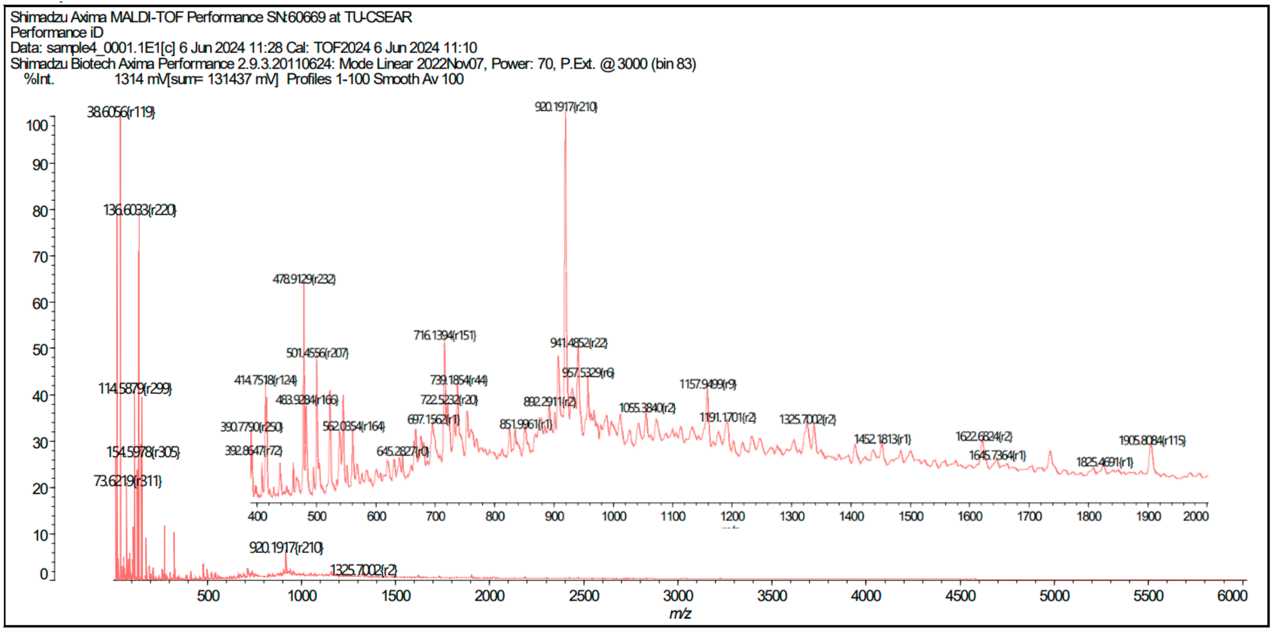

Hydroxypropylated starch low DS (HL)

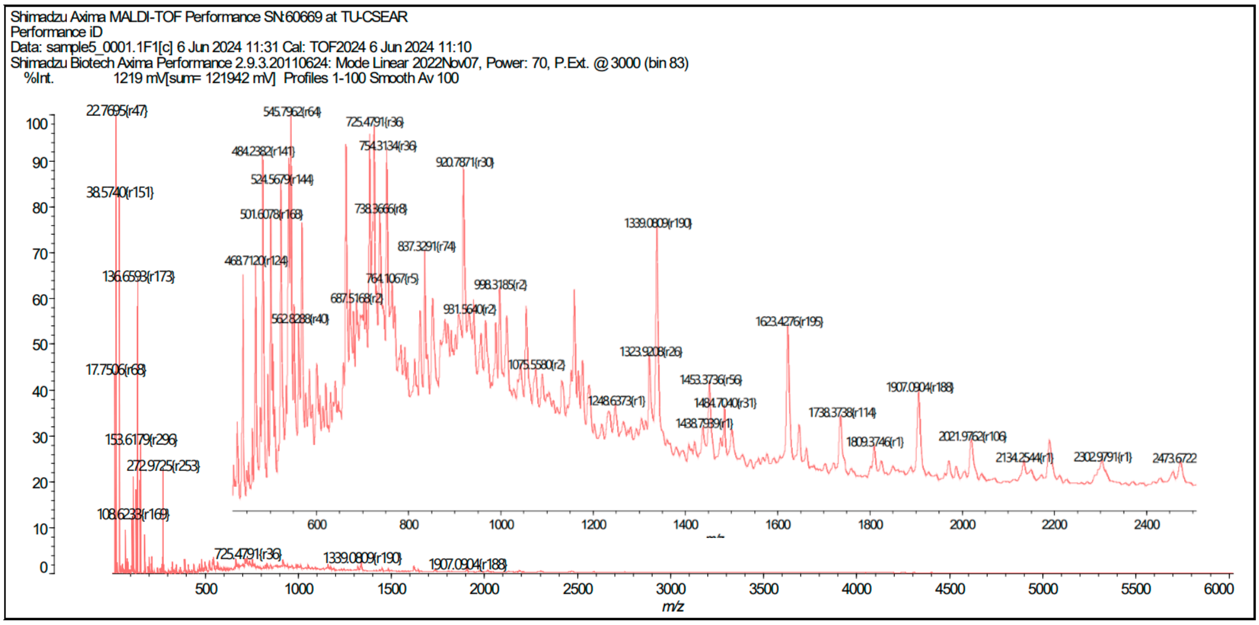

Hydroxypropylated starch high DS (HH)

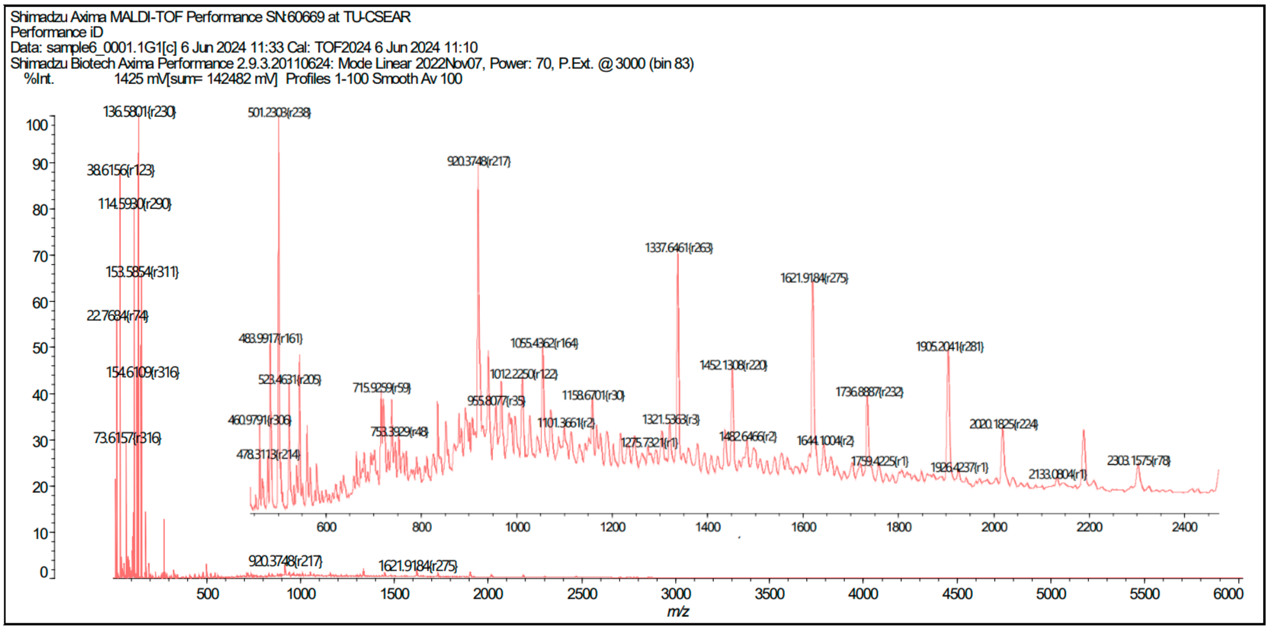

Native starch with ethyl maltol (NTE)

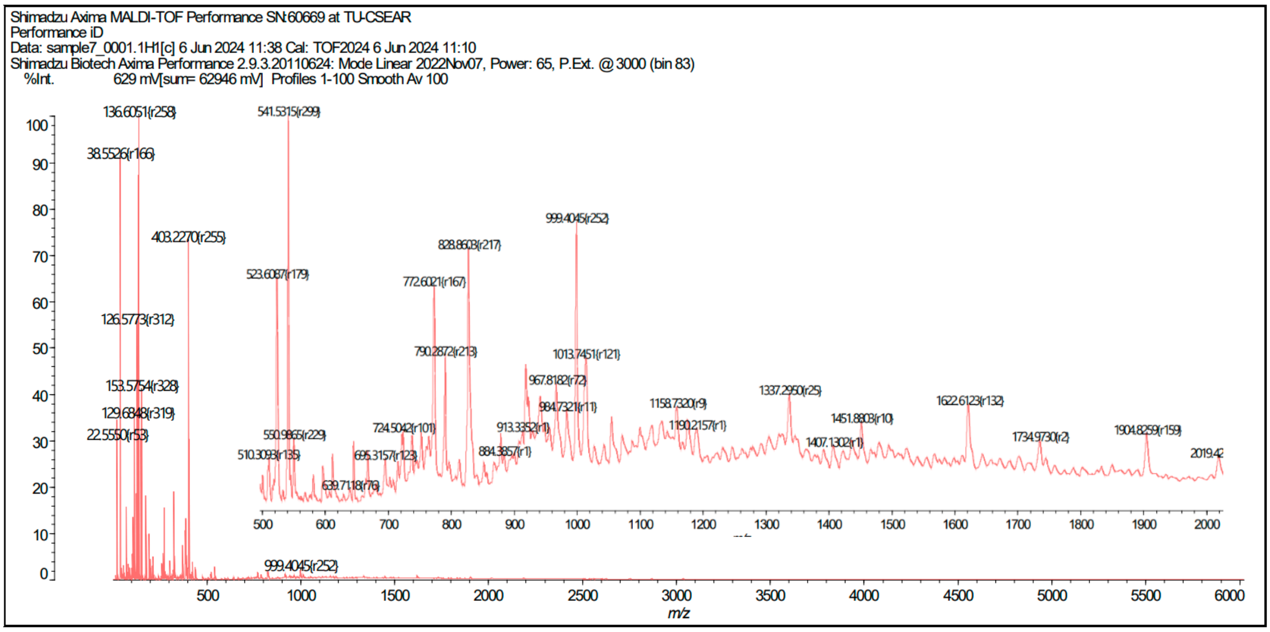

Octenyl succinated starch with ethyl maltol (OSE)

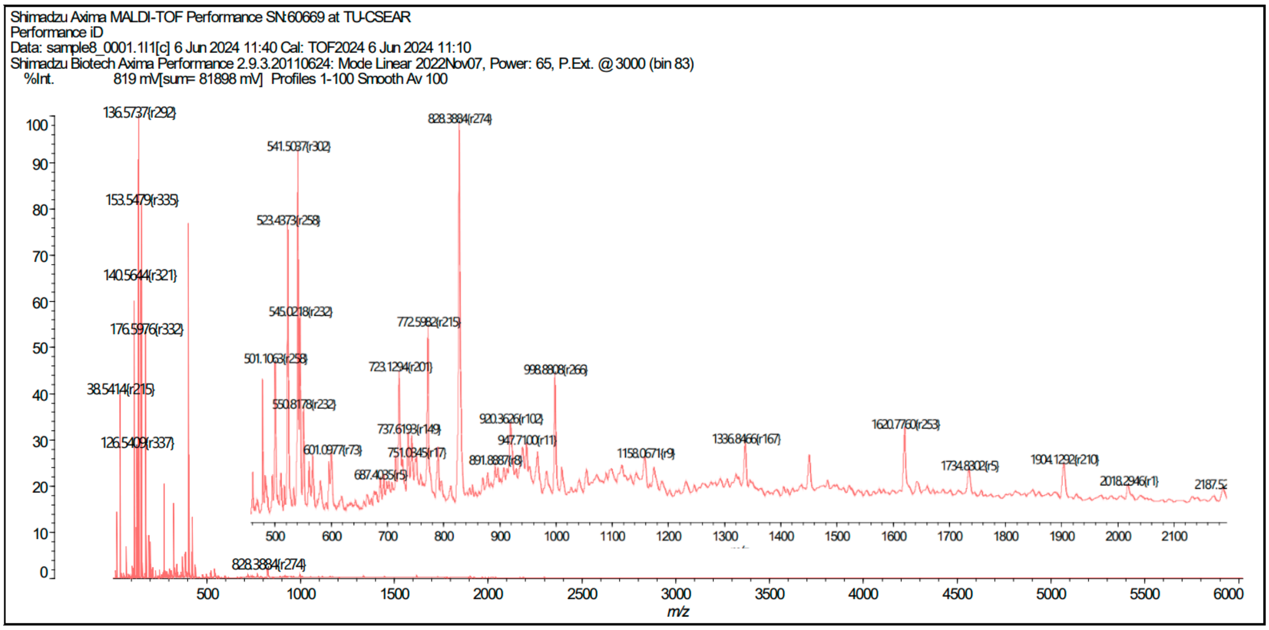

Acetylated starch with ethyl maltol (ACE)

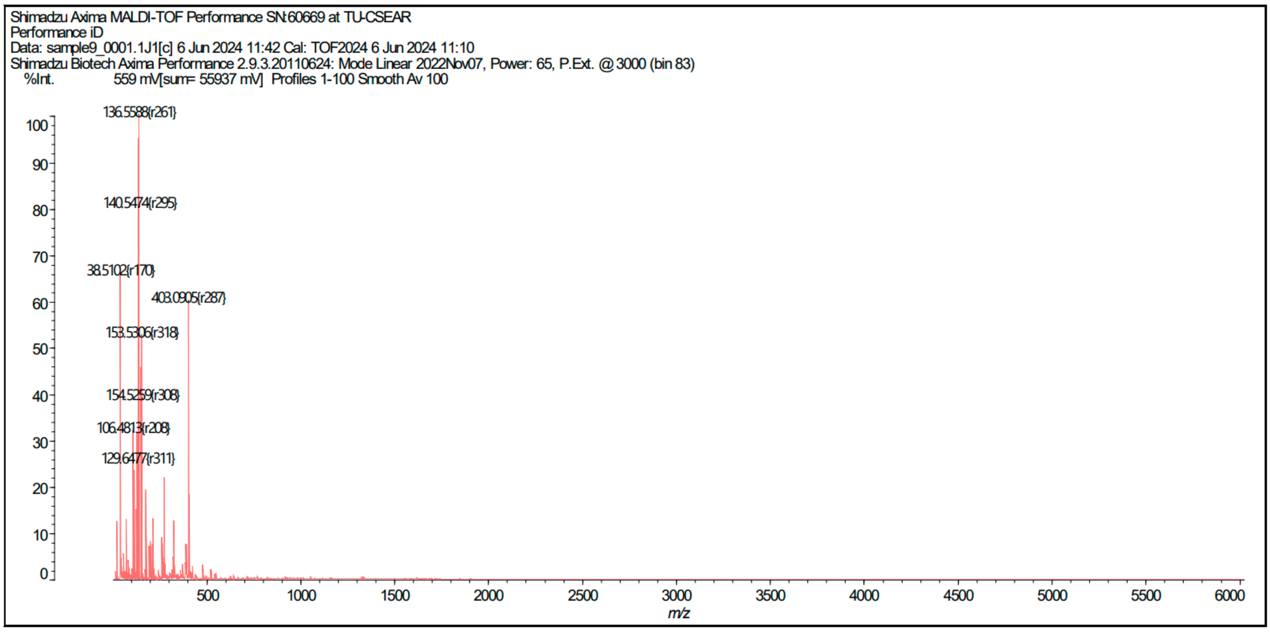

Hydroxypropylated starch medium DS with ethyl maltol (HME)

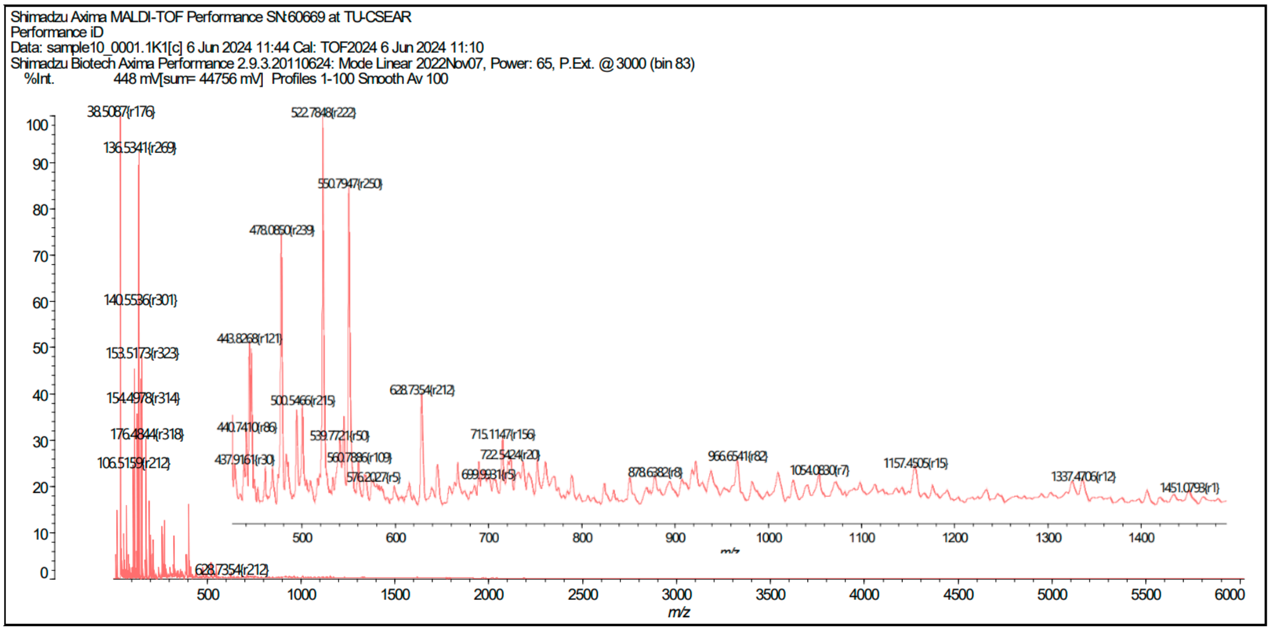

Hydroxypropylated starch low DS with ethyl maltol (HLE)

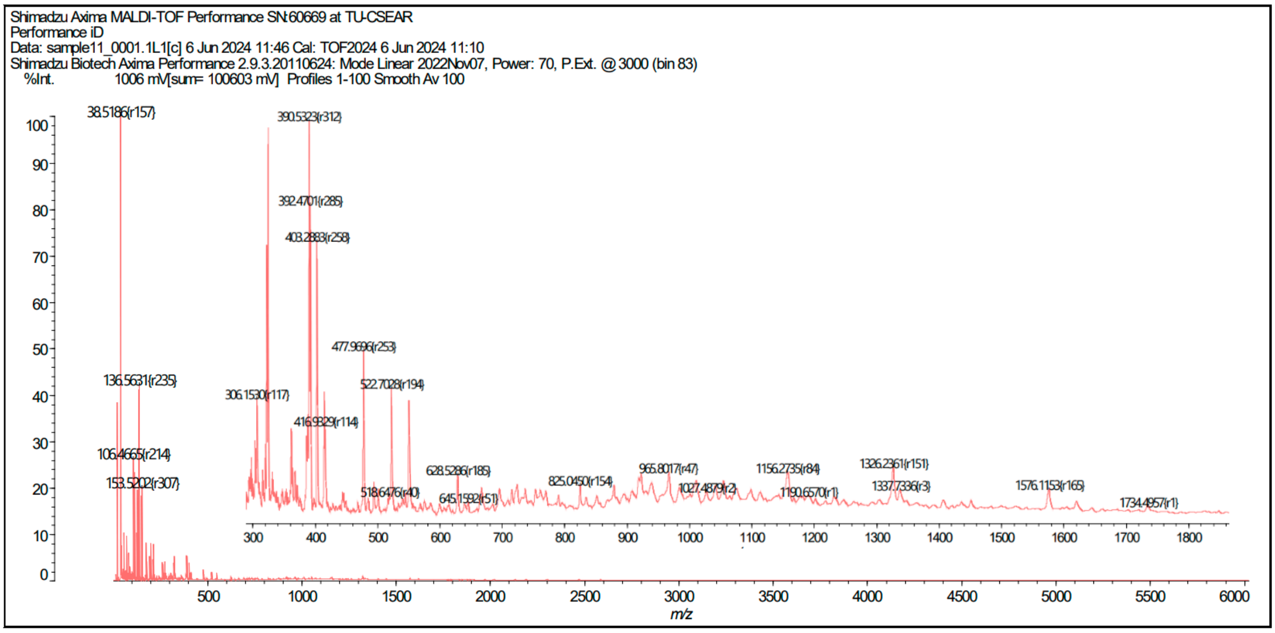

Hydroxypropylated starch high DS with ethyl maltol (HHE)

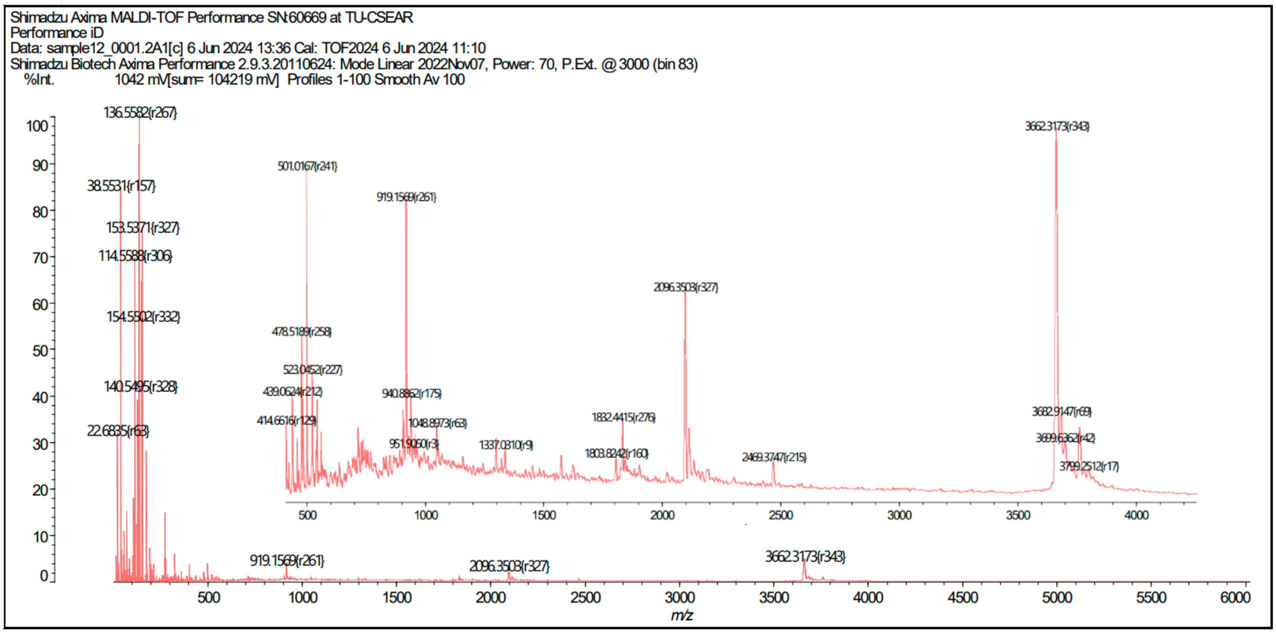

Native starch with maltol (NTM)

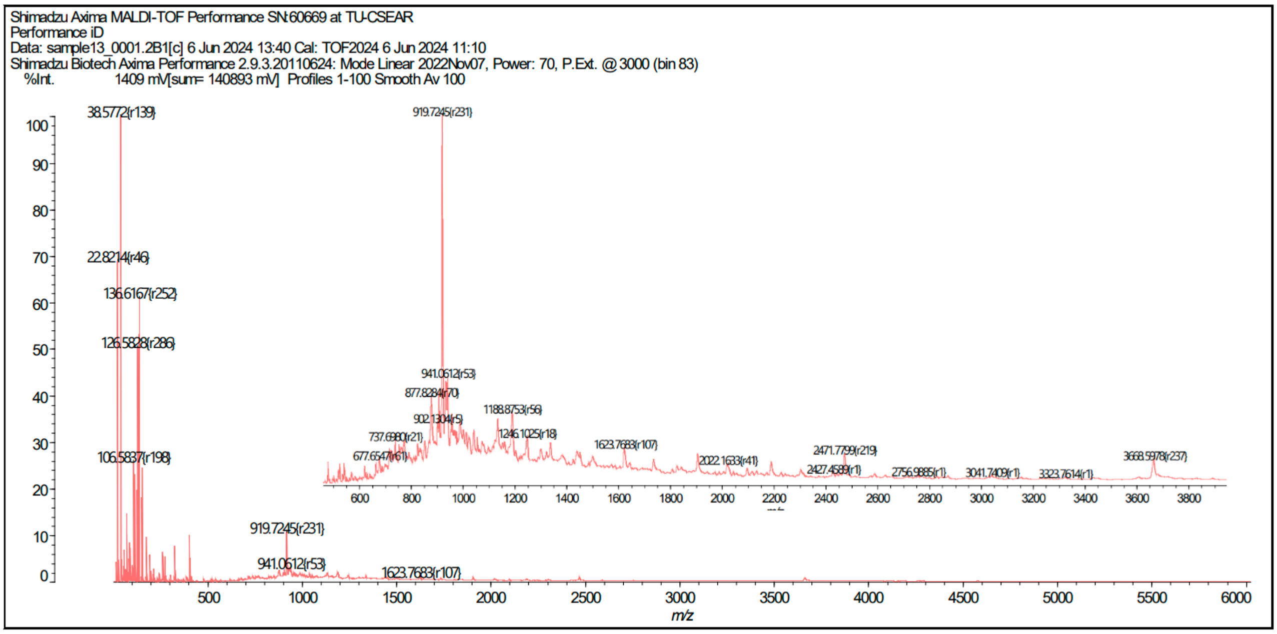

Octenyl succinated starch with maltol (OSM)

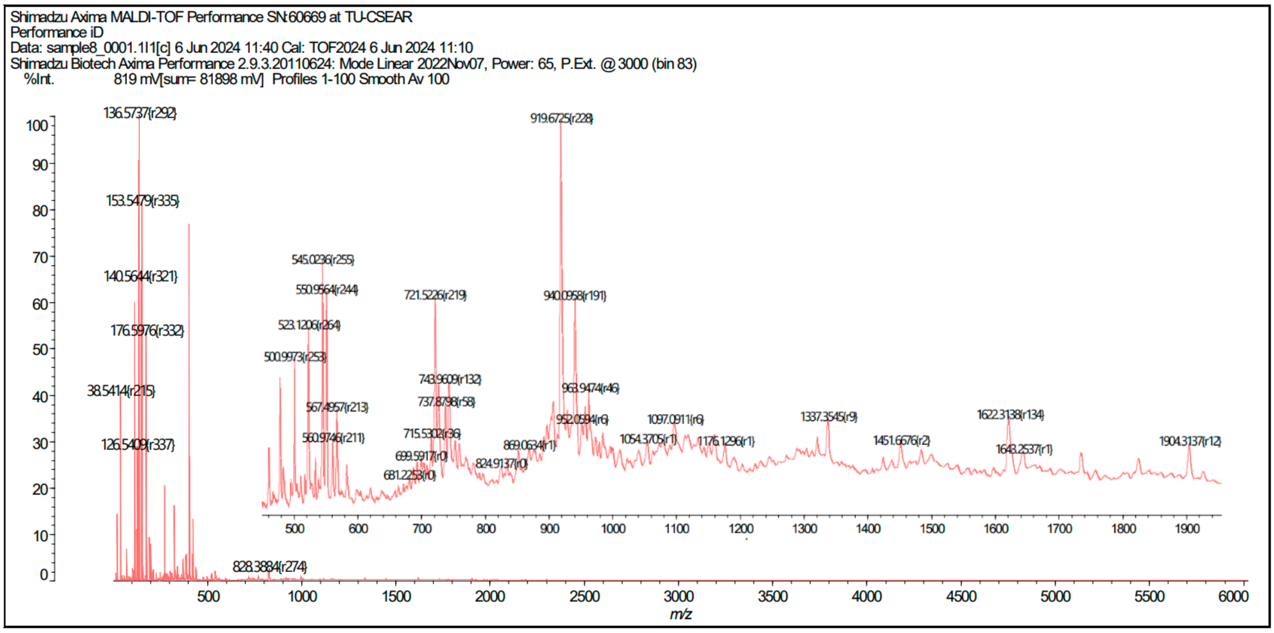

Acetylated starch with maltol (ACM)

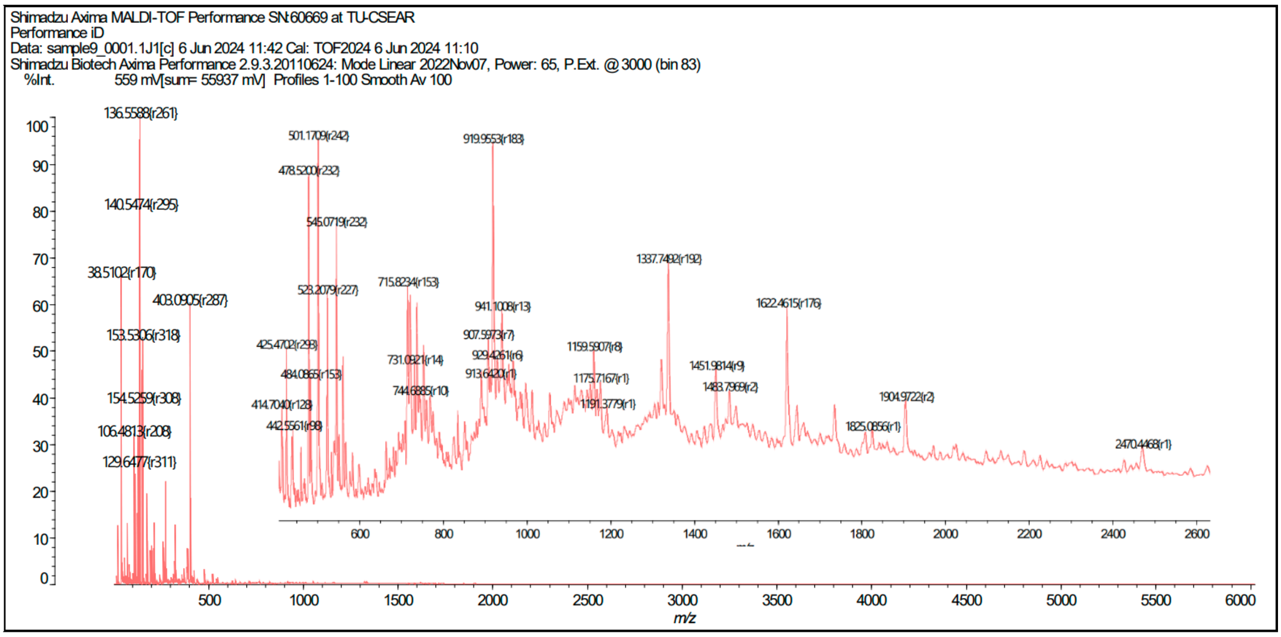

Hydroxypropylated starch medium DS with maltol (HMM)

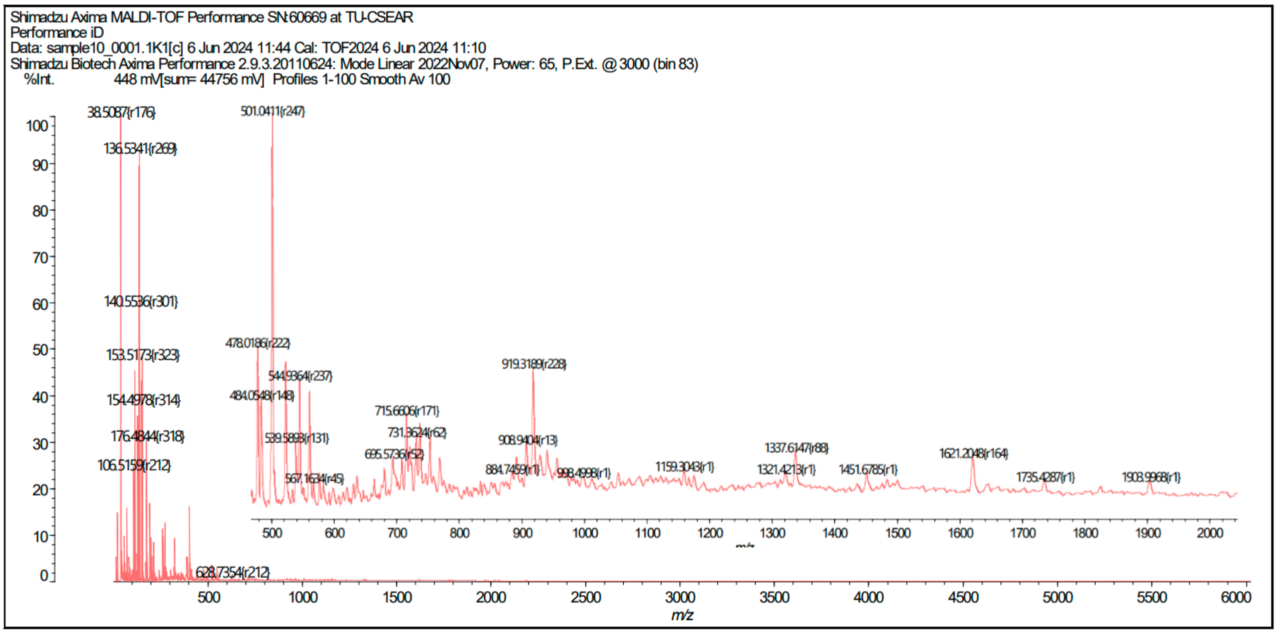

SupplementaryHydroxypropylated starch low DS with maltol (HLM)

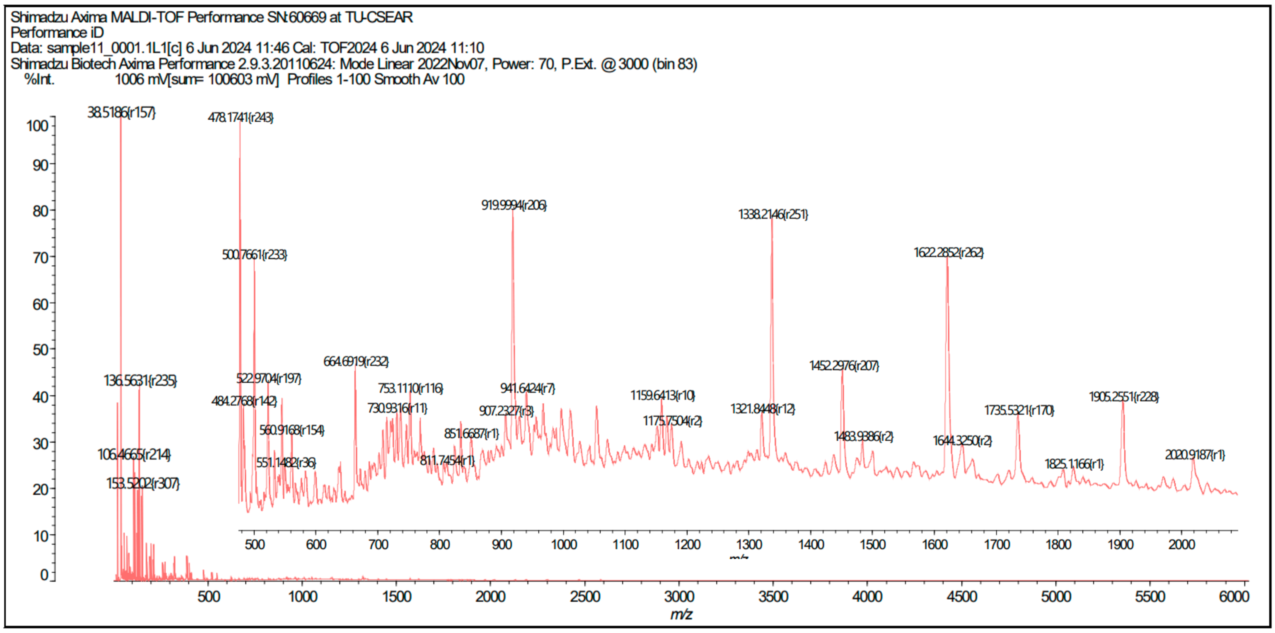

Hydroxypropylated starch high DS with maltol (HHM)

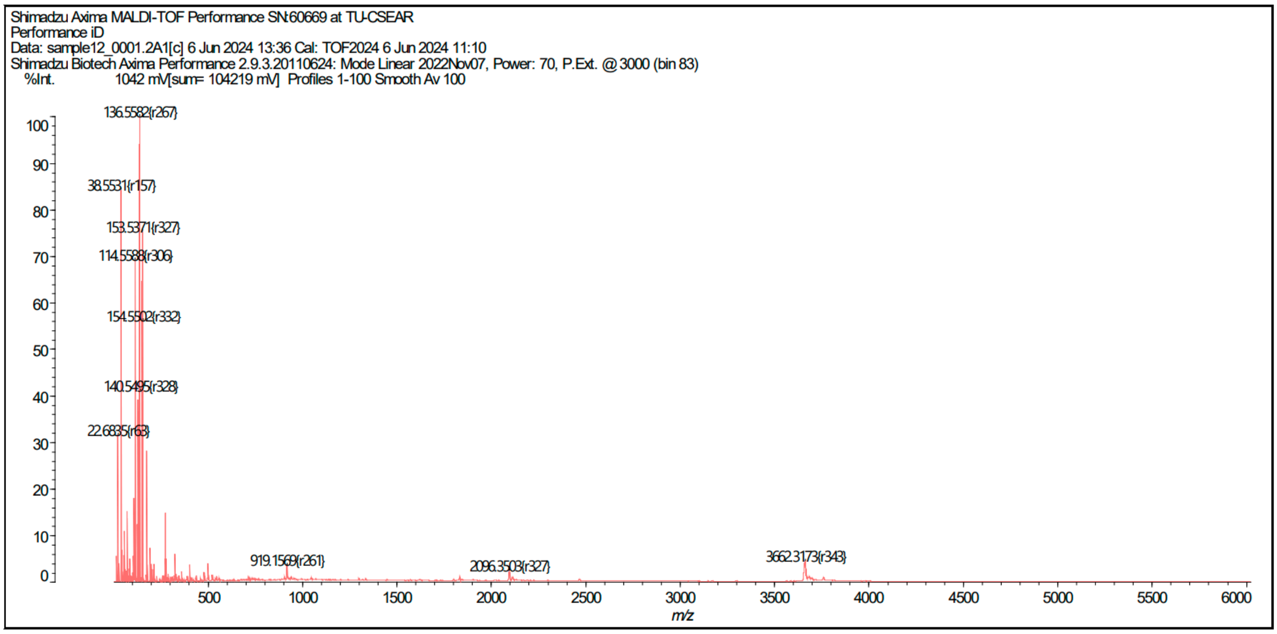

Fig. S3. Molecular weights of the native starch (NT), acetylated starch (AC), octenyl succinate (OS), hydroxypropylated low DS (HL), hydroxypropylated medium DS (HM), and hydroxypropylated high DS (HH) with ethyl maltol (E) and maltol (M) at 3% were determined by Matrix-Assisted Laser Desorption Ionization-Time of Flight Mass Spectrometry (MALDI-TOF MS) in reflectron mode.
